# A novel approach for yoga pose estimation based on in-depth analysis of human body joint detection accuracy

**DOI:** 10.7717/peerj-cs.1152

**Published:** 2023-01-13

**Authors:** Miral Desai, Hiren Mewada

**Affiliations:** 1 Department of EC Engineering, CSPIT, CHARUSAT, Anand, India; 2 Electrical Engineering Department, Prince Mohammad Bin Fahd University, AL Khobar, Saudi Arabia

**Keywords:** BlazePose, PCK, PDJ, Joint Detection, Pose Estimation

## Abstract

Virtual motion and pose from images and video can be estimated by detecting body joints and their interconnection. The human body has diverse and complicated poses in yoga, making its classification challenging. This study estimates yoga poses from the images using a neural network. Five different yoga poses, viz. downdog, tree, plank, warrior2, and goddess in the form of RGB images are used as the target inputs. The BlazePose model was used to localize the body joints of the yoga poses. It detected a maximum of 33 body joints, referred to as keypoints, covering almost all the body parts. Keypoints achieved from the model are considered as predicted joint locations. True keypoints, as the ground truth body joint for individual yoga poses, are identified manually using the open source image annotation tool named Makesense AI. A detailed analysis of the body joint detection accuracy is proposed in the form of percentage of corrected keypoints (PCK) and percentage of detected joints (PDJ) for individual body parts and individual body joints, respectively. An algorithm is designed to measure PCK and PDJ in which the distance between the predicted joint location and true joint location is calculated. The experiment evaluation suggests that the adopted model obtained 93.9% PCK for the goddess pose. The maximum PCK achieved for the goddess pose, *i.e.*, 93.9%, PDJ evaluation was carried out in the staggering mode where maximum PDJ is obtained as 90% to 100% for almost all the body joints.

## Introduction

Human pose estimation is one of the most challenging problems of computer vision. Human pose estimation involves localizing various body joints like shoulder, hip, elbow, ankle, knee, *etc.*, at the correct location of a static image or video data. Body joint detection and localization are carried out in two-dimensional coordinates (*x*, *y*) or three-dimensional coordinates (*x*, *y*, *z*). If the localization of joints is in 2D coordinates, then the estimated pose is considered 2D human pose estimation; otherwise, it is deemed to be 3D human pose estimation (Zheng et al., 2020). Representation of detected body joint localization on the input data consists of three approaches: (1) skeleton model, where body joints are detected in the point form and connected in the form of a line that creates limbs. (2) Shape model in which body parts are detected instead of body joints and represented in square boxes. (3) Mesh model represents the detected body parts as complete 3D volume. Therefore, the mesh model is used to represent 3D pose estimation. The skeleton model represents body-joint localization in the proposed approach (Desai & Mewada, 2021). The methodology to estimate human pose consists of the traditional method (Yang & Ramanan, 2012) and the deep neural network-based approach. Various deep neural networks like DeepPose (Toshev & Szegedy, 2014), OpenPose (Cao et al., 2018), Convolutional Pose Machine (Wei et al., 2016), Stacked Hourglass Network (Newell, Yang & Deng, 2016), BlazePose (Bazarevsky et al., 2020), *etc.*, are used to identify accurate body joint localization on the human pose. We used the BlazePose model to estimate body joints in the proposed article.

Estimated body joints are accurate if the location of estimated body joints is the same as actual joints. Accuracy is measured based on the percentage of detected joints (PDJ) as well as the percentage of corrected keypoints (PCK) (Dang et al., 2019). Human pose estimation is essential in various real-time applications like activity recognition, people tracking system, sports gaming, fitness application, yoga, asanas, *etc*.

In yoga, almost all important body joints of a human pose are activated. Therefore, yoga exercise needs proper knowledge and appropriate tutors. The recent trend is to follow the yoga pose online or through recorded videos. However, the evaluation of yoga following online or recorded sessions is complex. Furthermore, the human body has unusual poses in yoga, making it more challenging than other activities. Therefore, accurate localization of these body joints is of utmost importance for estimating the various yoga poses. In literature, different machine learning frameworks were adopted to detect body joints, including wearable sensor-based models (Wu et al., 2019; Puranik, Kanthi & Nayak, 2021), a Kinect model (Trejo & Yuan, 2018; Islam et al., 2017), OpenPose model (Cao et al., 2018), and computer vision-based models. Wu et al. (2019) presented artificial intelligence models using these sensor data for yoga pose recognition. However, the wearable sensor needs the attachment of the sensors to the body joints during yoga, and Kinect based approach needs a depth camera. Therefore, the sensor-based system is not convenient for users and is impractical. On the other hand, computer vision approaches are invasive and most suitable.

Accurate body joint detection plays a primary role in pose estimation and classification. The existing yoga pose identification techniques focus more on deep neural network-based classification models, and most literature evaluated the classification rate using confusion matrix parameters. Another alternative to assess the yoga pose classification is based on joint detection accuracy parameters viz. PCK, PDJ represents different method of yoga pose classification. This paper presents the adoption of a well-known machine learning algorithm to improve body joint detection from the image. According to the authors’ knowledge, quantitative evaluation of body-joint detection is unique among all literature. The main contributions of the paper are as follows:

• A well-known body-joints detection model, BlazePose, is adopted and trained from scratch for the localization of body joints in yoga poses.
• In contrast to the conventional pose estimation models, which use 18 body joints to represent a human pose, detecting 33 key points is proposed to achieve higher accuracy.
• True keypoints as the ground truth body joint is created using an open source image annotation tool named Makesense AI. All true keypoints of individual body joints are manually added and annotated on individual yoga poses.
• This article presents an in-depth analysis of each detected keypoint and a comprehensive study on various yoga poses using these key points. Finally, a quantitative evaluation and comparative analysis using PCK and PDJ are presented.

The article’s overall structure is as follows: Section 2 represents the related work carried out. The dataset used for testing and validating the proposed model is introduced in Section 3. The proposed model is explained in Section 4. The experimental setup and test results are discussed in Section 5. Finally, Section 6 gives the concluding remarks and further scope of application.

## Related work

Yoga plays a vital role in developing a balance between mental, physical, and spiritual states. The current situation of the COVID-19 pandemic creates maximum stress and anxiety worldwide (Sharma, Anand & Kumar, 2020). Regular yoga practice is essential in developing strong immunity and improving human beings’ psychological development. This leads to reduced stress and anxiety in the human body. The yoga technique is easy, flexible, and cost-effective as anyone can practice it at home only by learning basic yoga asana without any instrument. Yoga is the cumulative process of different types of “asana”. The term “asana” has been derived from the Sanskrit term, which means “posture” or “pose”. An asana is a different kind of body posture or poses like “Sukhasana (easy pose)”, “Naukasana (boat pose)”, “Dhanurasana (bow pose)”, “Bujangasana (Cobra pose)”, *etc*. Asanas help to enhance body flexibility by lubricating the muscles, joints, ligaments, and other body parts.

Literature proposed the work carried out by various researchers for automated yoga pose estimation during exercises and worked out. Thar, Winn & Funabiki (2019) proposed yoga pose classification methods for self-learning. Body joints are achieved using the OpenPose (Cao et al., 2018) approach, and 24 body joints are used to represent the complete human skeleton. This system can classify up to four yoga poses with 85% to 90% classification accuracy. However, the localization accuracy of body joints is not measured in the presented system.

Lin et al. (2021) introduced a self-practice yoga system for tracking the performance of body posture during exercises. The OpenPose model is used to detect 24 keypoints of body joints. The localization accuracy of body joints is measured according to the angle designed by the vectors *x* and *y* of respective joints. The mean and standard deviation of the measured angle is classified in the posture evaluation in three different classes. The presented approach does not measure the accuracy of body-joint localization as per the standard evaluation parameters of body joints.

Yamao & Kubota (2021) proposed a human pose recognition system using the PoseNet (Chen et al., 2017) model on the Pi (2015) Platform. The PoseNet model detected 18 body joints. The test results are presented, measuring the accuracy of pose recognition that demonstrated body parts’ movement. The body joints localization algorithm is based on the traditional angle method instead of standard evaluation parameters.

To guide and supervise yoga students for their correct yoga poses, Huang et al. (2020) have proposed “Miss yoga” a yoga assistant mobile application based on keypoint detection. The application is built based on the OpenPose model to detect correct body key points during yoga. Application estimates the keypoints and calculates the score by comparing estimated key points with corrected keypoints. The application also uses vocal instruction to assist yoga students in providing a cooperative environment. The proposed approach is more inclined to the accuracy of body posture rather than body-joint localization.

Trejo and Yuan proposed recognition of yoga poses using Kinect (Trejo & Yuan, 2018). Kinect technology comprises a depth camera sensor (Shotton et al., 2012). The proposed interactive system recognizes six different yoga poses, with the accuracy of the pose being measured using the mean and deviation of the joint location of concerned body parts. However, a depth camera sensor is not readily available for users.

The existing approach of yoga pose estimation represented a significant role in yoga pose classification. Furthermore, almost all current techniques used the OpenPose or PoseNet model to localize body joints and detect 18 to 24 body joints to recognize the whole body. However, we proposed a different human body joint detection approach and analyzed the pose estimation accuracy, using various yoga poses as the input. Furthermore, 33 body joints were identified from the BlazePose model representing the whole body. In the later stage of the proposed approach, the pose estimation accuracy is measured in the form of standard evaluation parameters, viz. PCK, PDJ, plays a vital role in classifying yoga poses.

## Dataset

**Figure 1:**
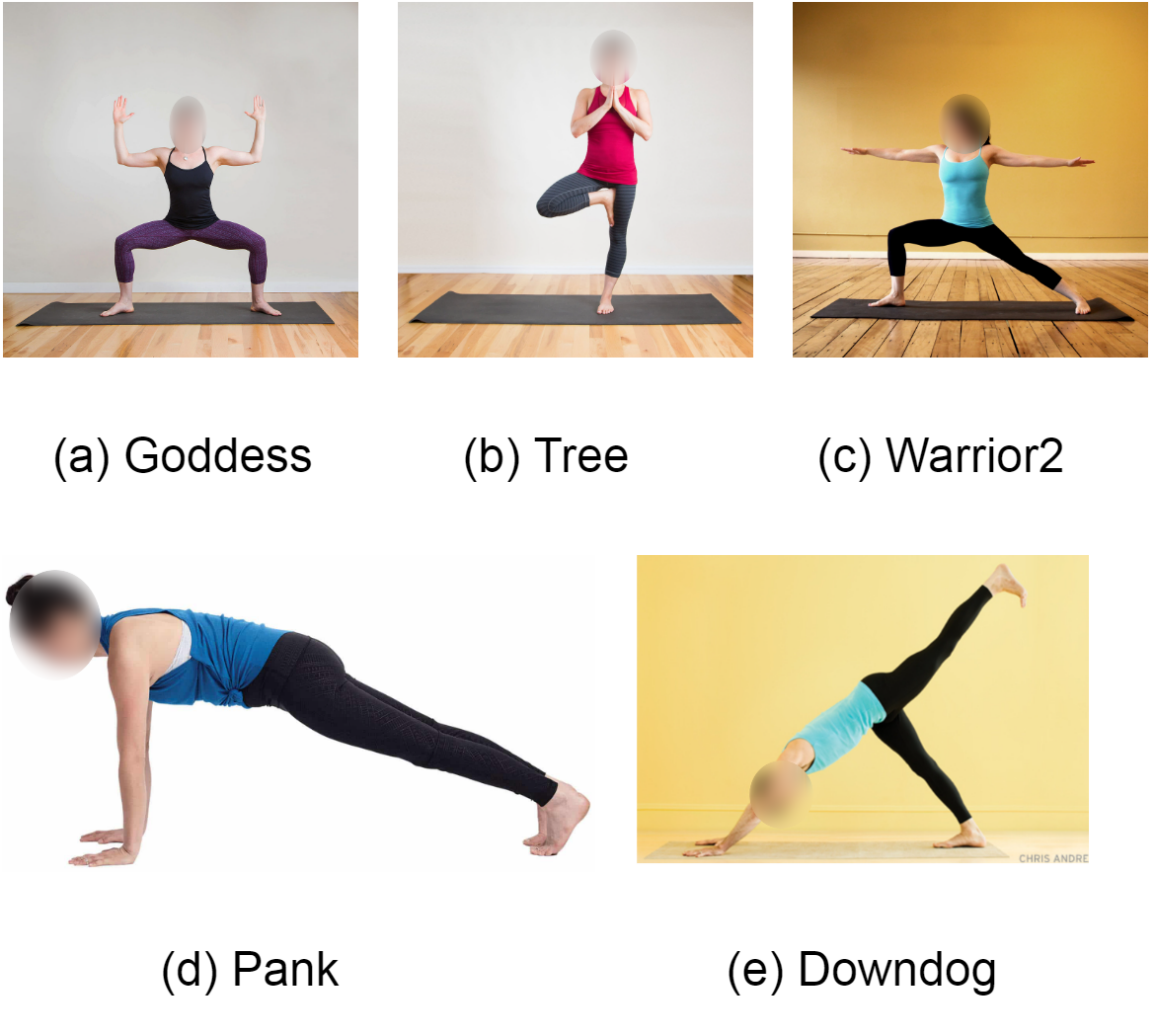
Sample images from dataset. Image source: https://www.kaggle.com/datasets/niharika41298/yoga-poses-dataset.

Any neural network’s input source is considered an image or video frame in detecting the keypoints to estimate the human pose. The images or video frame information is often represented in pixel values demonstrated in CSV files. Therefore, real-time images or video frames collected in CSV file is considered the dataset. The proposed system uses the Yoga Pose Dataset for further implementation (Pandit, 2020). The yoga poses datasets contain various yoga poses images like downdog poses, goddess poses, plank poses, tree poses, and warrior2 poses. The dataset contains 1,000 images of mentioned poses. A total of 70% of images of this dataset are used for training purposes, and the remaining 30% are used for testing purposes. Table 1 represents the summary of the Yoga Poses Dataset. Each image has a resolution of 300 × 300. The images in the dataset have different backgrounds with various skin colors and hair tones. The images are also captured from different camera angles and different lighting conditions. The sample images of all mentioned yoga poses are demonstrated in Fig. 1 Below.

**Table 1:** Summary of yoga poses dataset.

**Types of yoga poses**	**Total no. of images**	**Total no. of training images**	**Total no. of testing images**
Goddess	260	180	80
Tree	229	160	69
Warrior2	361	252	109
Plank	381	266	115
Downdog	320	223	97

## Proposed Approach

**Figure 2:**
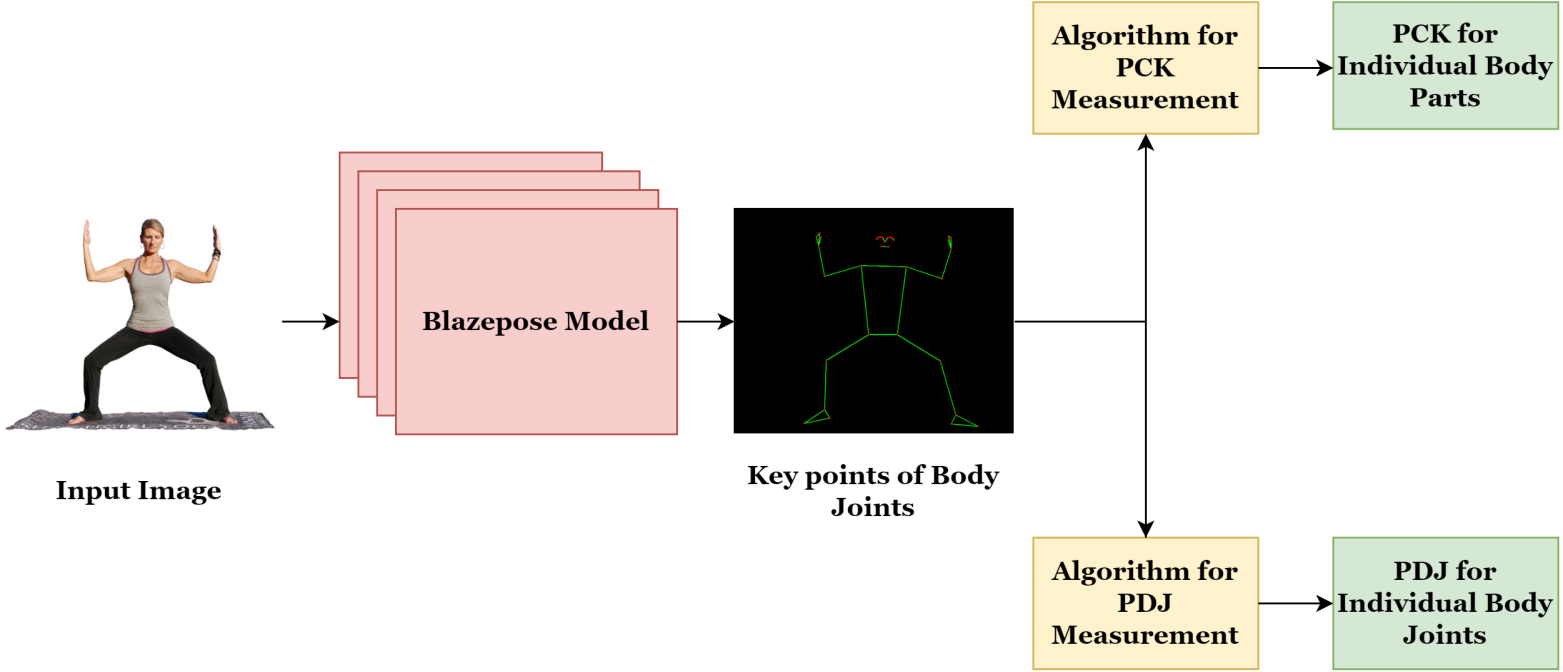
The functional flow of the proposed approach.

The proposed approach objective is to detect the number of human body joints and analyze the human pose estimation accuracy using percentage of correct keypoints (PCK) and percentage of detected joints (PDJ) evaluation parameters. As shown in Fig. 2, The overall approach is decomposed into two main phases. Firstly, detection of human body joints using the BlazePose model for all five yoga poses (for understanding, the general approach is exemplified with one pose goddess in Fig. 2). Secondly, accuracy analysis for all five yoga poses. In the second phase, the detected keypoints are validated using the annotation tool, and a discussion on joint-detection accuracy is presented.

### BlazePose model

**Figure 3:**
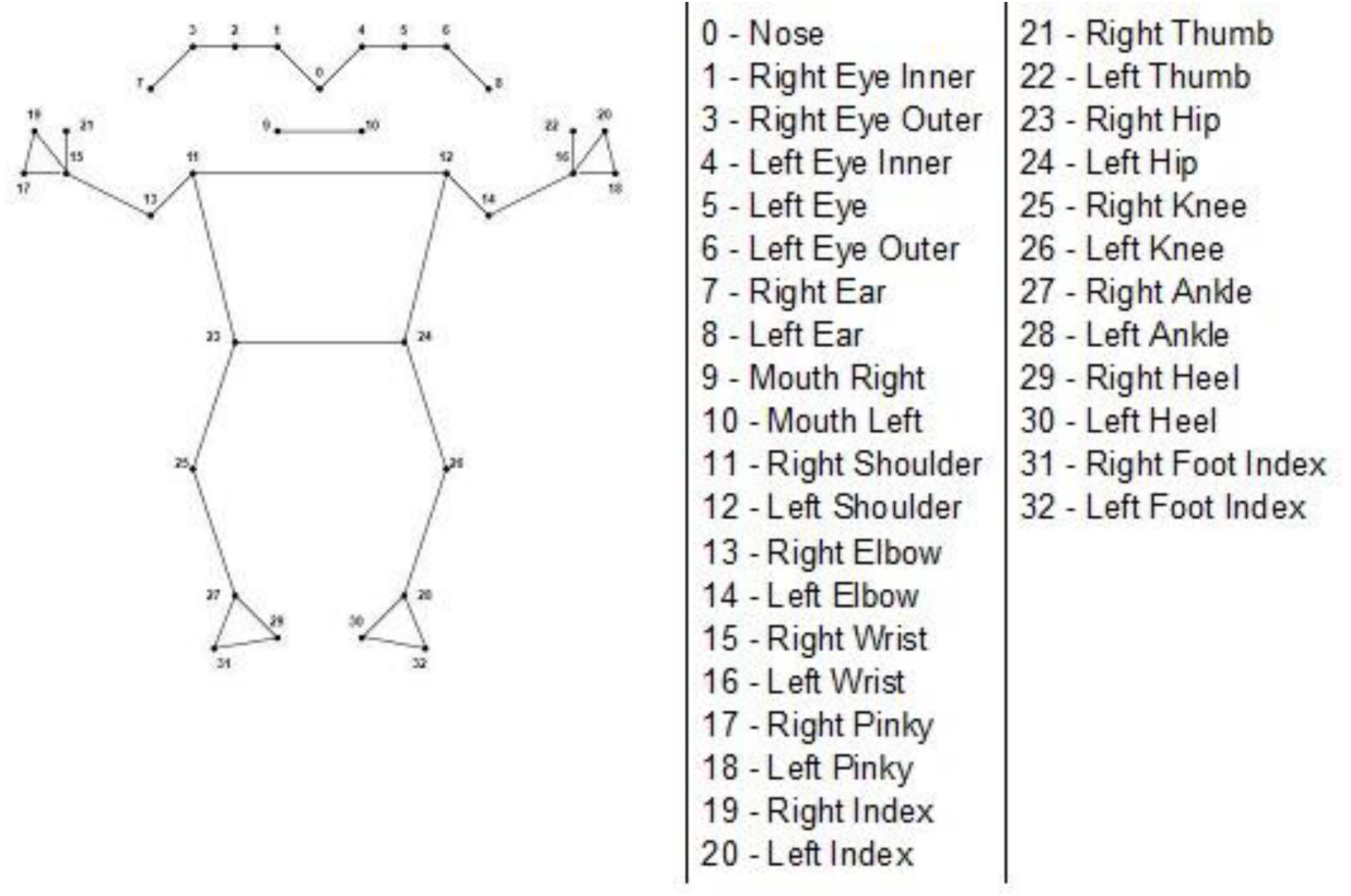
33 keypoints in BlazePose model.

Generally, human body joint detection is carried out using COCO topology, which uses 18 body joints to detect human pose (Lin et al., 2014). Representation of the viewpoint of human body joints as key points is carried out in the form of two different categories: (1) Person Centric (PC) and (2) Observer Centric (OC). The Person Centric viewpoint is considered the keypoint position decided concerning the person’s view. An Observer Centric viewpoint is regarded as a keypoint position determined concerning the observer’s view (Fu, Zhang & Huang, 2015). The BlazePose is another human pose estimation model developed by Google and presented at CVPR 2020 as an on-device real-time body pose tracking model. As shown in Fig. 3, the BlazePose model efficiently detects 33 human body joint landmarks in static images or videos, including the head, torso, arms, and legs. Moreover, it shows all 33 key points in depth per the person’s centric (PC) view. The BlazePose model can estimate and detect maximum body joints; it is used for dance poses and fitness applications.

**Figure 4:**
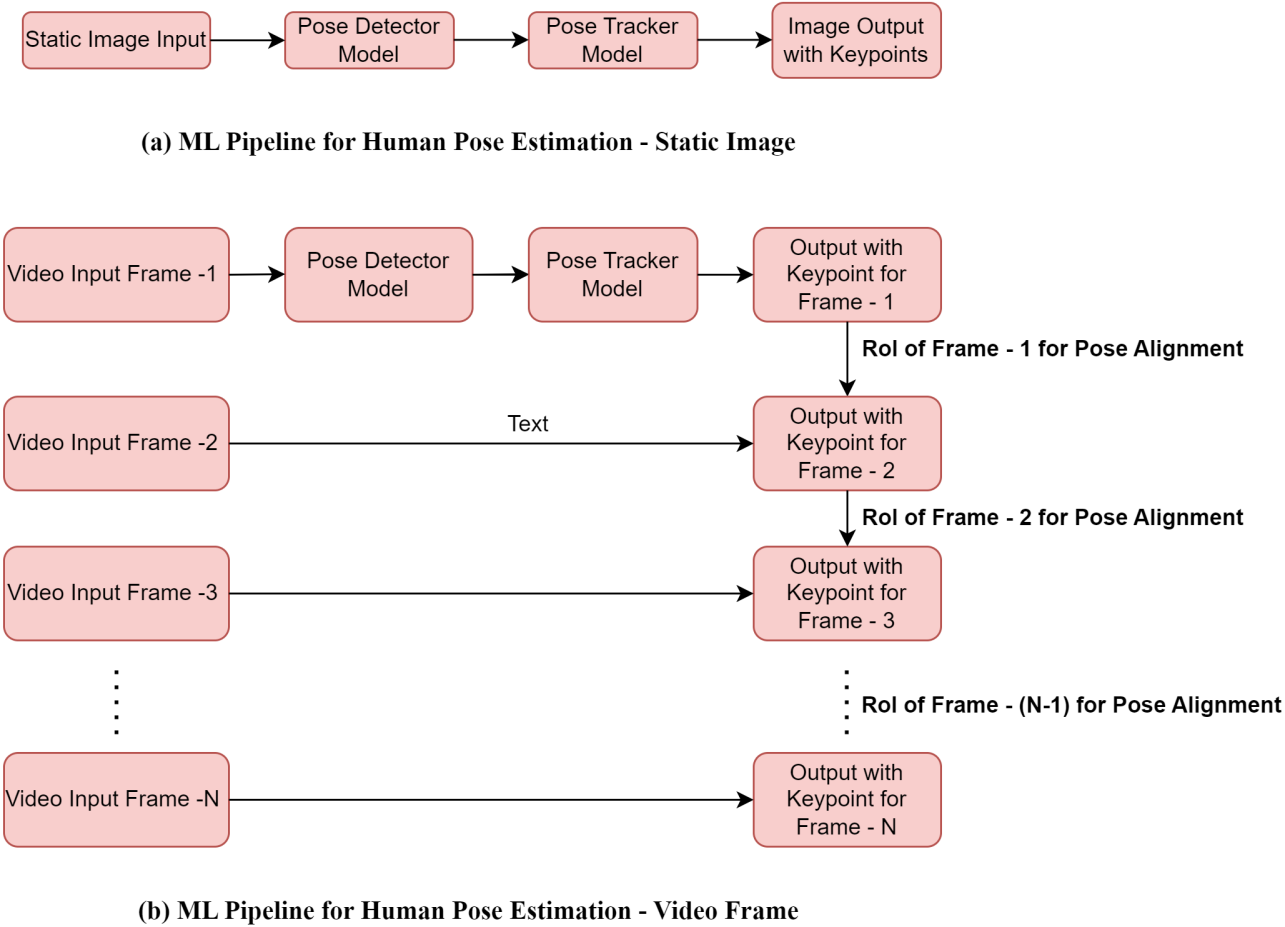
Machine learning pipeline approach for human pose estimation.

BlazePose model is an example of a machine learning pipeline (ML) approach for human pose tracking. The ML pipeline approach for human pose estimation combines pose detector and pose tracker. As shown in Fig. 4(A), the pose detector model can detect the human pose as the Region-of-Interest (RoI) from the static image or consisting of a video frame. The pose tracker model subsequently predicts 33 keypoints from the resultant RoI generated by the pose detector model. As shown in Fig. 4(B), the pose detector model only runs for the first frame and derives RoI for the video frame data. Once RoI is derived for the first frame, the pose tracker model runs for the same frame and identifies all 33 keypoints for the derived RoI. For the subsequent frames, RoI is derived from every previous frame, and the pose tracker model is applied to the respective frame for keypoints.

The human pose detection and tracking process of the ML pipeline must be speedy (in the form of a few milliseconds) for real-time applications like sports and yoga. The person’s face is the main target element to detect the pose speedily in the image and video. This is because the person’s face contains high-contrast variation and comparatively more minor variation than the appearance. It results from training a simple neural network to estimate the primary position of a person for the input data. The motivation for the Face detector training is taken from the Blazeface model (Bazarevsky et al., 2019), where a submillisecond neural face detection algorithm is carried out on the mobile platform. The model only detects the position of a single person within the image or video input. However, it cannot identify the position of multi persons as individuals. Once the face is detected, the estimated position of the remaining part of the human body is carried out from the inspiration of Leonardo da Vinci’s Vitruvian Man approach.

**Figure 5:**
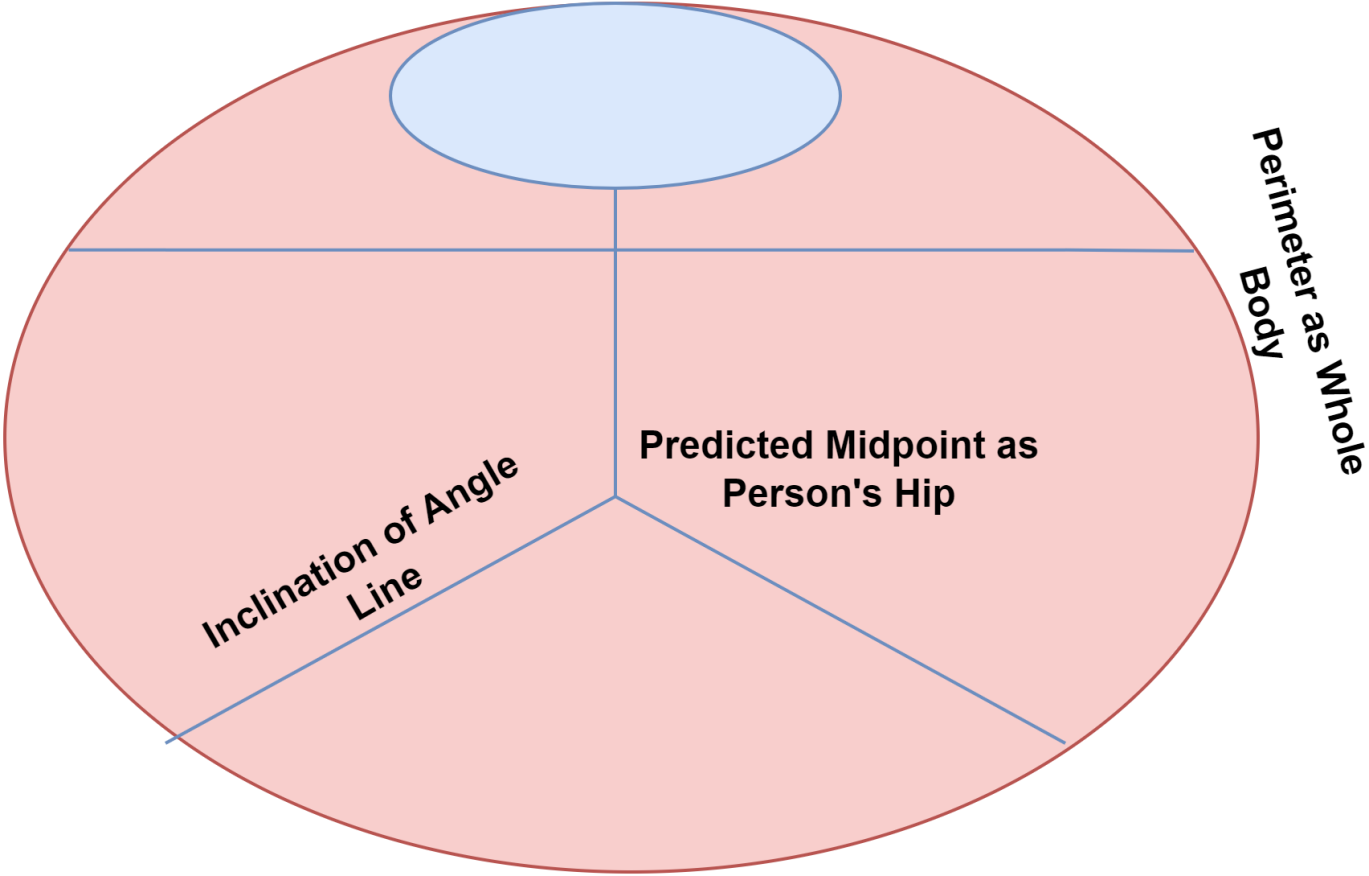
Leonardo da Vinci’s Vitruvian man approach.

In addition to face detection using the Blazeface model, Leonardo’s approach to estimating the body part’s position is based on the global centric circle. Here the center of the ring is the predicted midpoint of the person’s hip, the radius of the ring is the perimeter of the whole body, and the inclination of the angle line connects the shoulder and hip’s midpoint. The given approach even tracks the person’s position in complex cases like yoga asanas and fitness applications. Figure 5, shown below, illustrates the suggested method.

**Figure 6:**
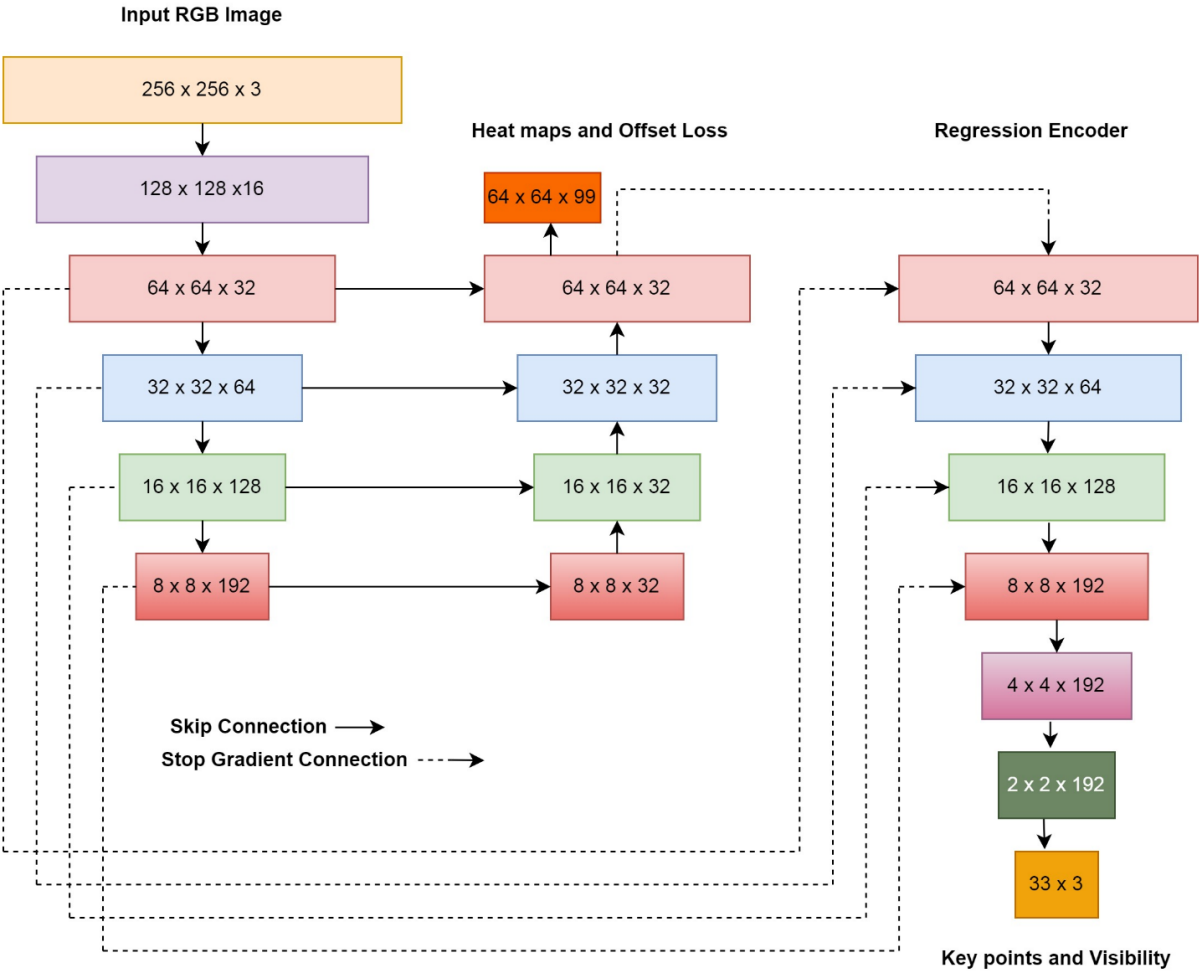
Tracking architecture for BlazePose model.

The tracking architecture of the BlazePose model is shown in Fig. 6. The overall training of the model is divided into two sections: heatmap and regression. Initially, the model trains the input image to identify the heatmap of the human body joints. In the later part of the model, this heatmap will be used as the supervised element for the regression encoder, which confers the body-joint location. The output of the model is 33 keypoints as human body joints. All 33 key points are represented as individual landmarks. The landmarks include (*x*, *y*, *z*), visibility, and presence. Here (*x*, *y*, *z*) indicates the position of individual keypoints in the image or frame. Visibility represents the probability of keypoints occluded by the object in the frame. Presence represents the probability of a keypoint in the frame. Thus, landmarks consist of 165 elements for individual keypoints.

In the proposed approach, the BlazePose algorithm is applied to input images for estimating the keypoints. It combines offset, heatmap, and regression to detect keypoints from the frame. First, the heatmaps are used effectively to control lightweight embedding; later, the regression encoder uses them. The skip connection provides a balance between the low and high-level features. The model localizes and estimates the keypoints on the input image and also records localization using *x* and *y* coordinates in the CSV file. These key points are considered as predicted joints for the further procedure. Summarization of localization of keypoints using the BlazePose model is represented in Algorithm 1.

**Table:** 

**Algorithm 1: Localization of Keypoints using Blazepsoe Model**
**Input**: Set of RGB Images — P
Set of BlazePose Landmarks — L
**Output**: Set of Resultant Images R imposed with pose Landmark L
Set of BlazePose coordinates in train.csv file.
1. Initialize supported python libraries for BlazePose model
2. Initialize BlazePose landmark — L
3. Initialize Input Image path (P)
4. Define path for Resultant Images (R)
5. **For** P Images **do**
Train BlazePose model to estimate the localization of all body joints
Save the resultant images (R)
**End**
6. Record Blazepsoe coordinates x,y in train.csv
7. Return {train.csv, Resultant Images R}

### Image annotation and true keypoint estimation using AI tool

Estimation of accuracy needs the true position of body joints in the image. Unfortunately, no such database is available specifically for various yoga poses. In the proposed approach, we used the Makesense AI image annotation tool (Prats Cristia, 2021) to mark the ground truth joints of the pose within images. Make sense AI is the open-source and platform-independent image annotation tool used for labeling the keypoints in the object detection environment of images. Before starting the labeling process, the labels are imported manually for the respective objects. The Makesense AI tool has three different labeling methods: Point Form, Line Form, and Polygon Form. The keypoint detection is labeled in point form. However, the area and volume of the object can be marked in the line form or the polygon form. Keypoints and resultant regions can be extracted from the image in the form of *x* and *y* coordinates of the respective pixels with reference to the size of the image. The pixel information is exported in a CSV file. All 33 keypoints are loaded manually as labels for further annotation in the proposed approach. After fixing the labels, input images are imported to the annotation tool. Once image input is given, all the visible joints are manually annotated according to the labels. After completing the annotation, all recorded key points are exported to a CSV file. These key points are considered actual joints for further quantitative parameters evaluation.

### Analysis of pose estimation accuracy

The accuracy measurement for human pose estimation is carried out by identifying the correct position of keypoints or estimating the correct position of human body parts. Different evaluation parameters include PCP, PCK, PDJ, AP, AR, OSK, *etc*. PCK and PDJ parameters are almost identical, except the multiplication factor to achieve the threshold are different, as shown in Eq. (1) and Eq. (2). In the proposed approach, we focus on PDJ and PCK evaluation parameters to measure the accuracy of the human pose.

#### Percentage of detected joints (PDJ)

In the initial stage of human pose estimation, the percentage of corrected parts (PCP) is considered the evaluation parameter for pose estimation, which measures the accuracy of detected limbs (body parts). However, the major limitation of PCP is failing to measure accuracy for short limbs. Therefore, a PDJ evaluation parameter is introduced by Munea et al. (2020). PDJ is the detected joint found correct if the mean distance between the predicted joint and ground through joint lies within certain threshold limits. However, the threshold is defined as follows:

(1) \begin{eqnarray*}\text{Threshold as PDJ@0.2}& =& {|}\text{Predicted Joint}-\text{Ground Truth Joint}{|}\nonumber\\\displaystyle & & \lt 0.2\ast \text{Torso Diameter}.\end{eqnarray*}

Here Torso Diameter is the distance between two opposite body joints of the torso, *i.e.,* the distance between the right hip and left shoulder.

Threshold rates can be changed by changing the fraction value from 0.1 to 0.4 to achieve better accuracy of PDJ. The higher value of PDJ expressed a better model.

#### Percentage of correct keypoints (PCK)

The percentage of correct key points defined as estimated keypoints is considered valid if the distance between predicted joints (achieved by training the model) and ground truth joints lies within the boundary of certain threshold limits. However, the threshold is defined as follows:

(2) \begin{eqnarray*}\text{Threshold as PCK@0.2}& =& {|}\text{Predicted Joint}-\text{Ground Truth Joint}{|}\nonumber\\\displaystyle & & \lt 0.2\ast {\mathrm{Threshold}}_{\mathrm{Max}}.\end{eqnarray*}

Here ThresholdMax Torso Height is considered a side with a maximum length of the outer rectangle covering ground truth body joints (Shamsafar & Ebrahimnezhad, 2018). The higher value of PDJ expressed a better model.

Keypoint estimation accuracy is proposed by developing an algorithm using PCK and PDJ as evaluation parameters. The PCK and PDJ algorithm is developed in the MATLAB environment, where predicted and ground truth joints are used to compute the localization accuracy of body joints. Algorithm 2 presents accuracy parameters PCK and PDJ calculation.

**Table:** 

**Algorithm 2: Computation of accuracy parameters PCK and PDJ**
**Input**: Set of Predicted body joints — R
Set of Real body joints — K
Set of Body joints Name — A
Set of Body Part Name — B
**Output**: PCK for individual body part B and average PCK
PDJ (0.1 → 0.4) for individual body Joints A
1. Initialize all body joints (as per Blazepsoe Model) as set C
2. Compute PCK:
{PCK_B_, Mean}← {R, K,C, B};
Return {PCK_B_, Mean}
3. Initialize Reference joint pair as set D
Compute PDJ:
{PDJ_0.1 → 0.4_}← {R, K, D, C, A};
Return {PDJ_0.1 → 0.4_}

## Experimental setup & test results discussion

The experimental setup of the proposed approach is a stepwise process. The first step is the keypoints localization using the BlazePose model and actual keypoint estimation using the AI tool. The second step is the computation of accuracy, *i.e.,* PCK and PDJ. The resultant PCK and PDJ achieved for the BlazePose model are compared with the OpenPose model. The OpenPose model identifies 18 keypoints as per the COCO dataset notation. The yoga dataset input image is given to the OpenPose model to determine the location of 18 keypoints. These keypoints are considered the predicted keypoints. Later, the Image annotation tool is used to localize the ground truth joints as per the COCO dataset notations. For specific yoga poses like downdog pose and plank pose, we observed that some body parts are not visible due to complex posture. In the ground truth localization process, non-visible keypoints are replicated by visual keypoint localization content. For example, for the downdog pose, if the right shoulder body part is not visible in the image and the left shoulder body part is visible. Localization of the left shoulder is replicated for the right shoulder body part. The computation algorithm finds PCK and PDJ for various yoga poses for OpenPose models. Table 2 represents the summary of keypoint localization operating BlazePose and OpenPose model for different yoga poses. It is significantly observed that the OpenPose model fails to localize three keypoints for warrior2 posture, six keypoints for plank pose, and 12 keypoints for downdog pose.

**Table 2:** Summary of keypoint localization using BlazePose and OpenPose model for various yoga poses.

**Yoga poses**	**Model name**	**Targeted keypoints**	**Detected keypoints**	**Missing keypoints**
Goddess	BlazePose	33	33	0
	OpenPose	18	18	0
Tree	BlazePose	33	33	0
	OpenPose	18	18	0
**Warrior2**	BlazePose	33	33	0
	**OpenPose**	18	**15**	**3**
**Plank**	BlazePose	33	33	0
	**OpenPose**	18	**12**	**6**
**Downdog**	BlazePose	33	33	0
	**OpenPose**	18	**6**	**12**

**Notes.**

(Significance of Bold Text): For Warrior2 Yoga pose, OpenPose Model detected only 15 keypoints and missing 3 keypoints as compared BlazePose Model. For Plank Yoga pose, OpenPose Model detected only 12 keypoints and missing 6 keypoints as compared BlazePose Model. For Downdog Yoga pose, OpenPose Model detected only 6 keypoints and missing 12 keypoints as compared BlazePose Model.

**Figure 7:**
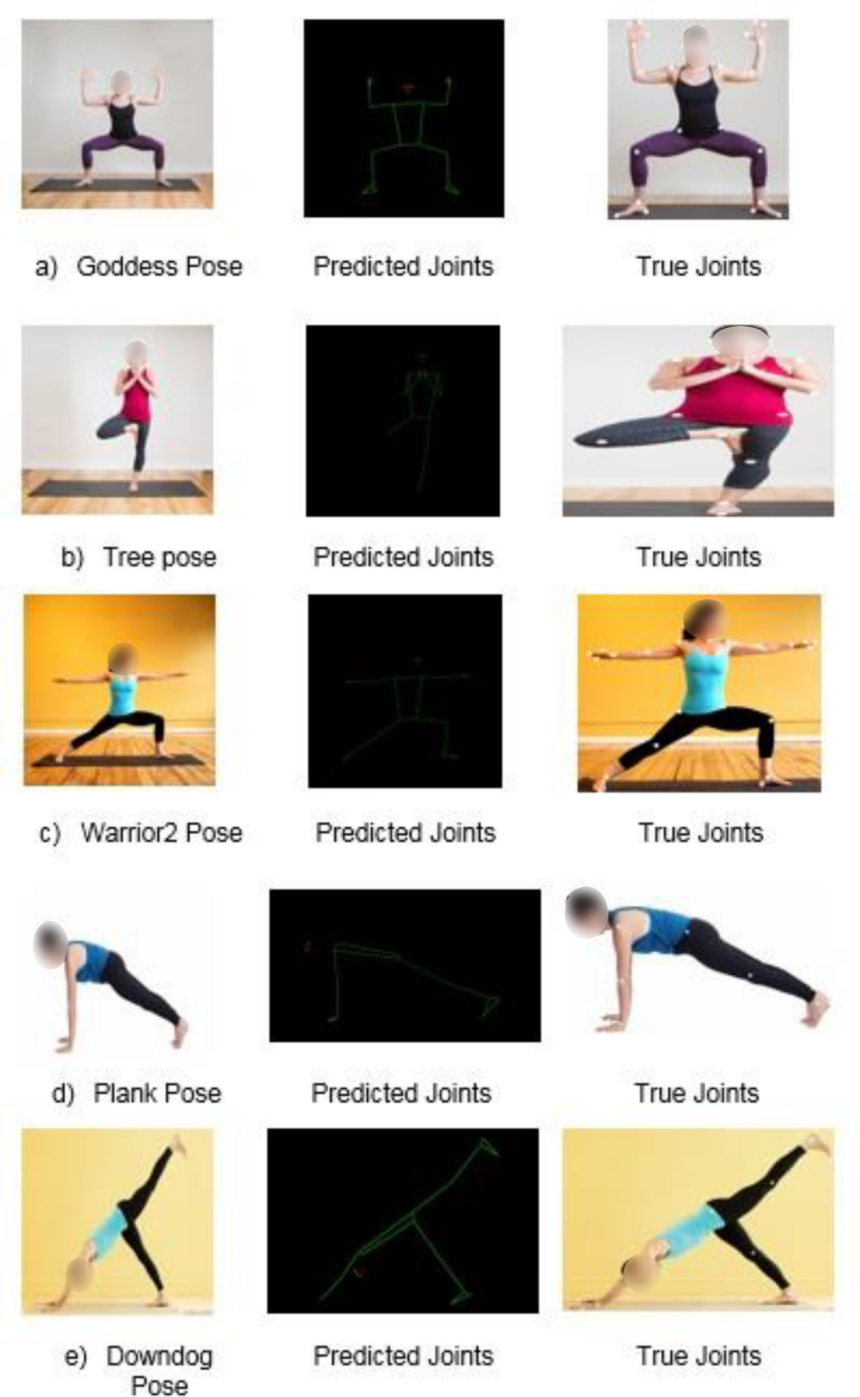
Yoga poses with predicted joints and true joints location obtained using proposed model. Image source: https://www.kaggle.com/datasets/niharika41298/yoga-poses-dataset.

The ground truth localization of body joints achieved from the image annotation tool and the estimated localization of body joints acquired using the BlazePose model for individual yoga poses are shown in Fig. 7. For all five yoga poses, the BlazePose model predicted the joint location; however, in some wired poses, viz. downdog and plank, a few of the body joints are missing. For these missing joints, the model has been trained so that, first, it predicts the location of the present joint and takes the projection of the present symmetry joint. Here the model detects the location of the projected point, and finally, it localizes the body joint, which is not visible in the input image. As shown in Fig. 7(D), it is demonstrated that only the left shoulder is visible in the input image for the plank pose. At the same time, the right shoulder is not visible. In this case, the BlazePose model first predicts the location of the left shoulder and then takes the same parameter’s projection to detect the right shoulder’s location. However, as shown in Fig. 7(E), a few of the body joints of a plank yoga pose is not visible and therefore cannot be detected through the image annotation tool. The missing joint locations are considered using symmetry in the actual joint location analysis phase. For example, in the plank pose, the right shoulder is not visible in the input image. However, the left shoulder is visible in the input image. So with consideration of the left and right shoulders being symmetrical to each other, we considered the localization of the left shoulder as the localization of the right shoulder in the process of ground truth localization.

Table 3 represents the PCK evaluation achieved from the model, which runs individually for various yoga poses. Quantitative information described in Table 3 shows that the overall body pose is achieved by estimating several body parts from the Nose to Foot Index. Table 3 shows that the goddess pose obtained maximum PCK, *i.e.,* 93.9%, whereas the downdog pose obtained minimum PCK, *i.e.,* 69.7%. Table 3 shows the accuracy order for estimating yoga poses as goddess, tree, warrior2, plank, and downdog. The downdog pose replicates joint locations using symmetry without body joints in the ground truth localization.

**Table 3:** Individual percentage of correct keypoints (PCK) on various yoga poses using BlazePose model.

**Body parts**	**Yoga poses**				
	**Goddess**	Tree	Warrior2	Plank	Downdog
Nose	100	100	100	100	100
Eye Inner	100	100	50	0	100
Eye	100	100	50	100	100
Eye Outer	100	100	50	100	100
Ear	100	100	50	100	0
Mouth	100	100	50	100	100
Shoulder	100	100	100	100	100
Elbow	50	100	100	100	100
Wrist	100	100	100	50	100
Pinky	100	100	100	50	0
Index	100	0	100	50	0
Thumb	100	0	100	50	0
Hip	50	100	100	50	100
Knee	100	100	100	100	100
Ankle	100	100	100	50	100
Heel	100	50	50	50	100
Foot Index	100	50	50	100	0
**Mean**	**93.9**	81.8	78.8	72.7	69.7

**Notes.**

(Significance of Bold Text): The Goddess Yoga pose achieve highest mean PCK as 93.9% among all other Yoga Poses.

**Figure 8:**
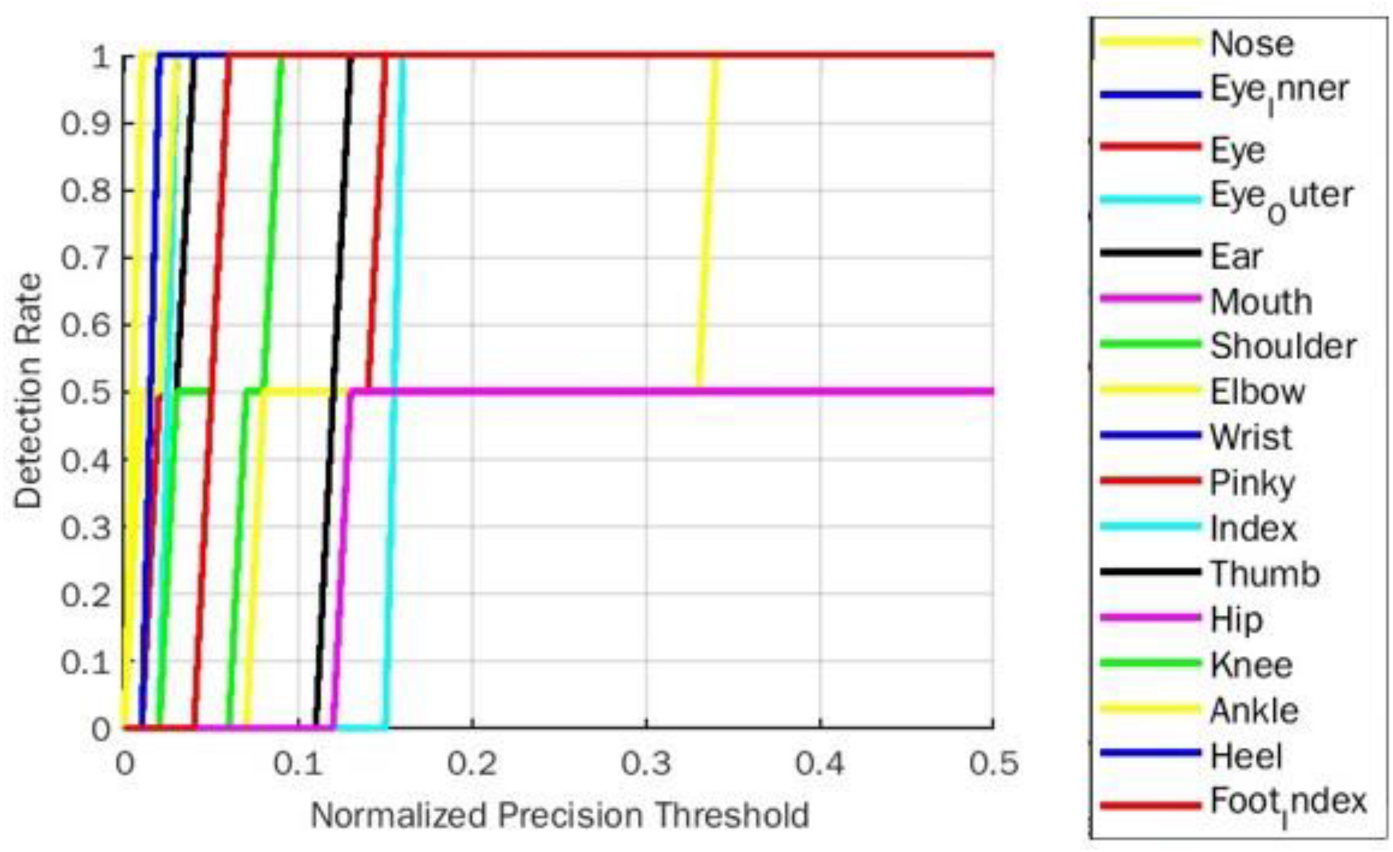
PDJ0.1_0.4 Detection rate on goddess yoga pose for BlazePose.

The PDJ0.1 →0.4 evaluation parameter for goddess yoga pose for BlazePose is represented in Table 4. As per Table 4 representation, the detection rate of a body part elbow was 50% initially with a threshold of 0.1, and it improved significantly to 100% PDJ at a 0.4 threshold. Among all estimated body parts, only the hip part gives 50% PDJ. The remaining parts achieved 100% in PDJ0.4. Figure 8 shows the PDJ0.1 →0.4 detection rate on goddess yoga pose for the BlazePose model.

**Table 4:** Percentage of detected joints (PDJ) on goddess yoga pose for BlazePose.

**Body parts**	**Goddess**			
	PDJ@0.1	PDJ@0.2	PDJ@0.3	PDJ@0.4
Nose	100	100	100	100
Eye Inner	100	100	100	100
Eye	100	100	100	100
Eye Outer	100	100	100	100
Ear	100	100	100	100
Mouth	100	100	100	100
Shoulder	100	100	100	100
Elbow	**50**	**50**	**50**	**100**
Wrist	100	100	100	100
Pinky	**0**	**100**	100	100
Index	**0**	**100**	100	100
Thumb	**0**	**100**	100	100
Hip	0	50	50	50
Knee	100	100	100	100
Ankle	100	100	100	100
Heel	100	100	100	100
Foot Index	100	100	100	100

**Notes.**

(Significance of Bold Text): The Pinky, Index and Thumb body parts improvise PDJ rate from 0% to 100% at different PDJ Level. The Elbow body part improvise PDJ rate from 50% to 100% at different PDJ Level. (Significance of Blue Text): The Hip body part indicate PDJ rate of only 50% due to improper visibility of this body part in Goddess Yoga Pose.

**Figure 9:**
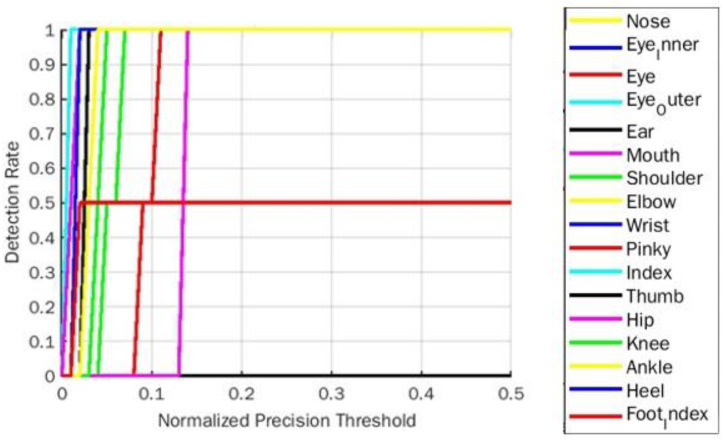
PDJ0.1_0.4 detection rate on tree yoga pose for BlazePose.

The PDJ0.1 →0.4 for tree yoga pose for BlazePose is shown in Table 5. Body parts index, and thumb results in 0% PDJ rate as these two parts are almost missing in the ground truth localization of tree pose. The heel and the foot index parts achieve 50% PDJ in the final PDJ stage. The remaining parts gained 100% in PDJ0.4. Figure 9 shows the PDJ0.1 →0.4 detection rate on tree yoga pose for the BlazePose model.

**Table 5:** Percentage of detected joints (PDJ) on tree yoga pose for BlazePose.

**Body parts**	**Tree**			
	PDJ@0.1	PDJ@0.2	PDJ@0.3	PDJ@0.4
Nose	100	100	100	100
Eye Inner	100	100	100	100
Eye	100	100	100	100
Eye Outer	100	100	100	100
Ear	100	100	100	100
Mouth	100	100	100	100
Shoulder	100	100	100	100
Elbow	100	100	100	100
Wrist	100	100	100	100
Pinky	**50**	**100**	100	100
Index	0	0	0	0
Thumb	0	0	0	0
Hip	**0**	**100**	100	100
Knee	100	100	100	100
Ankle	100	100	100	100
Heel	50	50	50	50
Foot Index	50	50	50	50

**Notes.**

(Significance of Bold Text): The Hip body part improvise PDJ rate from 0% to 100% at different PDJ Level. The Pinky body part improvise PDJ rate from 50% to 100% at different PDJ Level. (Significance of Blue Text): The Heel and Foot Index body parts indicate PDJ rate of only 50% due to improper visibility of these body parts in Tree Yoga Pose. (Significance of Red Text): The Index and Thumb body parts indicate PDJ rate of 0% due to invisibility of these body parts in Tree Yoga Pose.

**Figure 10:**
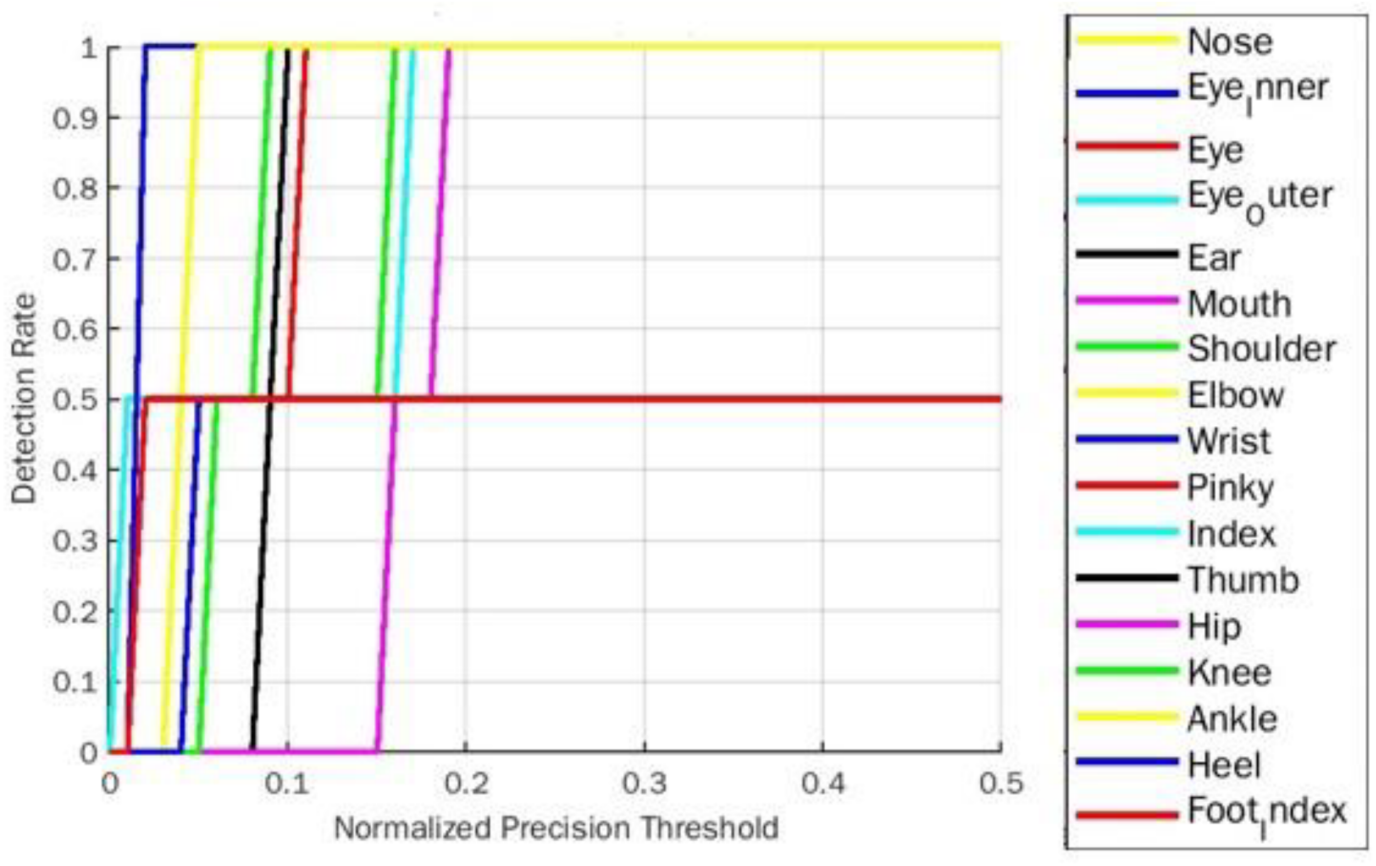
PDJ0.1_0.4 detection rate on warrior2 yoga pose for BlazePose.

The PDJ0.1 →0.4 for the warrior2 yoga pose is demonstrated in Table 6. As per Table 6, a few body parts achieve 50% of the PDJ rate, covering the eye, ear, mouth, heel, and foot index from the overall body parts. The remaining body parts achieved 100% as PDJ0.4. Figure 10 shows the PDJ0.1 →0.4 detections rate on warrior2 yoga pose for the BlazePose model.

**Table 6:** Percentage of detected joints (PDJ) on warrior2 yoga pose for BlazePose.

**Body parts**	**Warrior2**			
	PDJ@0.1	PDJ@0.2	PDJ@0.3	PDJ@0.4
Nose	100	100	100	100
Eye Inner	50	50	50	50
Eye	50	50	50	50
Eye Outer	50	50	50	50
Ear	50	50	50	50
Mouth	50	50	50	50
Shoulder	100	100	100	100
Elbow	100	100	100	100
Wrist	100	100	100	100
Pinky	**50**	**100**	100	100
Index	**0**	**100**	100	100
Thumb	100	100	100	100
Hip	**0**	**100**	100	100
Knee	**50**	**100**	100	100
Ankle	100	100	100	100
Heel	50	50	50	50
Foot Index	50	50	50	50

**Notes.**

(Significance of Bold Text): The Index and Hip body parts improvise PDJ rate from 0% to 100% at different PDJ Level. The Pinky and Knee body parts improvise PDJ rate from 50% to 100% at different PDJ Level. (Significance of Blue Text): The Eye Inner, Eye, Eye Outer, Ear, Mouth, Heel & Foot Index body parts indicate PDJ rate of only 50% due to improper visibility of these body parts in Warrior2 Yoga Pose.

**Figure 11:**
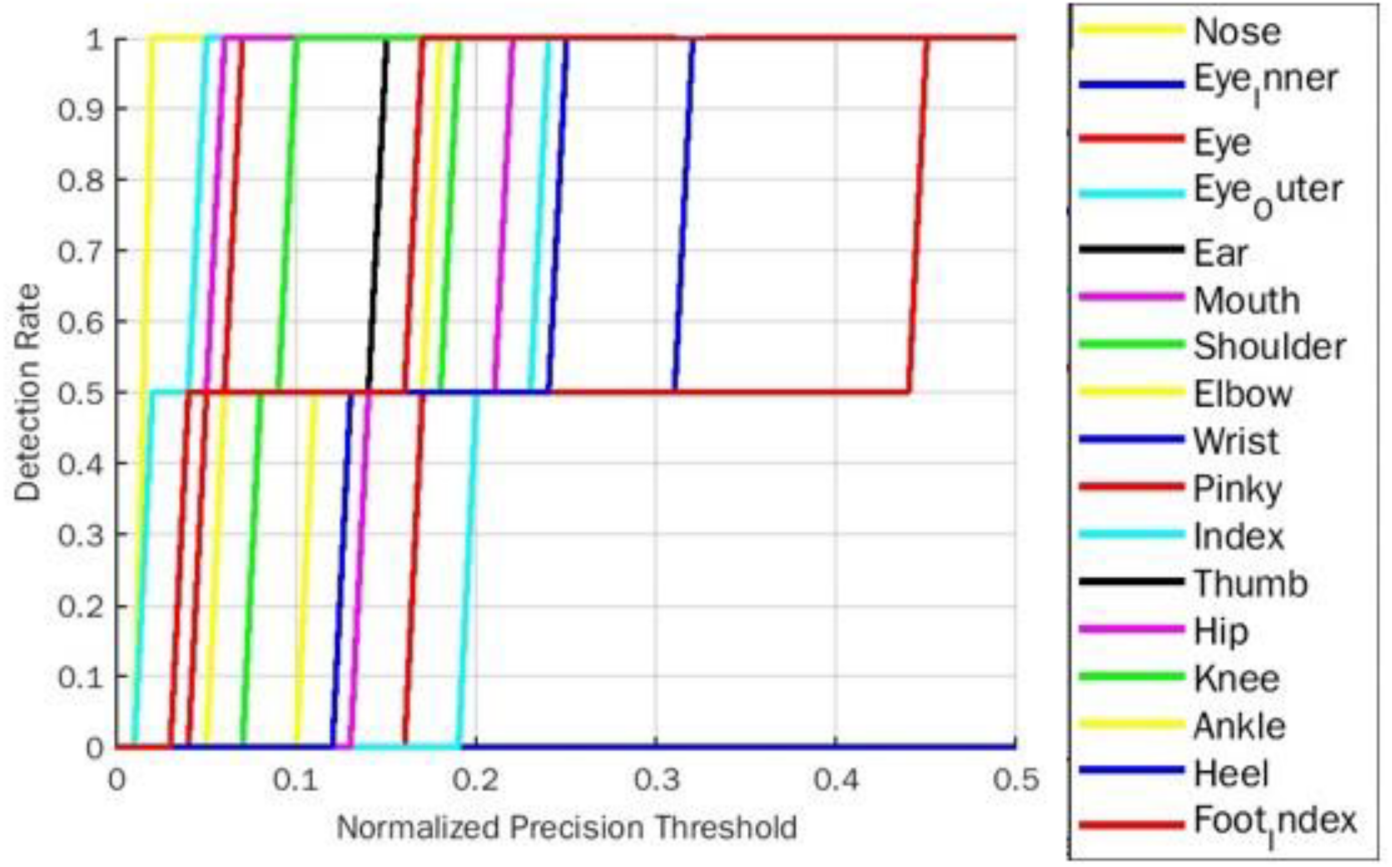
PDJ0.1_0.4 detection rate on plank yoga pose for BlazePose.

The plank yoga pose achieves a 100% PDJ rate for nose as body parts. The remaining body parts identify 50% as the PDJ rate. PDJ0.1 →0.4 for plank yoga pose is demonstrated in Table 7. As shown in the table, the eye inner is not visible from the pose therefore, we obtained PDJ as 0 for Eye Inner. Figure 11 shows the PDJ0.1 →0.4 detection rate on Plank Yoga Pose for the BlazePose model.

**Table 7:** Percentage of detected joints (PDJ) on plank yoga pose for BlazePose.

**Body parts**	**Plank**			
	PDJ@0.1	PDJ@0.2	PDJ@0.3	PDJ@0.4
Nose	100	100	100	100
Eye Inner	0	0	0	0
Eye	100	100	100	100
Eye Outer	100	100	100	100
Ear	**0**	**100**	100	100
Mouth	100	100	100	100
Shoulder	**50**	**100**	100	100
Elbow	**50**	**100**	100	100
Wrist	**50**	**50**	**50**	**100**
Pinky	0	50	50	50
Index	**0**	**50**	**100**	100
Thumb	**0**	**50**	**100**	100
Hip	**0**	**50**	**100**	100
Knee	100	100	100	100
Ankle	**0**	**50**	**100**	100
Heel	**0**	**50**	**100**	100
Foot Index	**50**	**100**	100	100

**Notes.**

(Significance of Bold Text): The Ear, Index, Thumb, Hip, Ankle and Heel body parts improvise PDJ rate from 0% to 100% at different PDJ level. The Shoulder, Elbow, Wrist and Foot Index body parts improvise PDJ rate from 50% 100% at different PDJ level. (Significance of Blue Text): The Pinky body part indicate PDJ rate of only 50% due to improper visibility of this body part in Plank Yoga Pose. (Significance of Red Text): The Eye Inner body part indicate PDJ rate of 0% due to invisibility of this body part in Plank Yoga Pose.

**Table 8:** Percentage of detected joints (PDJ) on downdog yoga pose for BlazePose.

**Body parts**	**Downdog**			
	PDJ@0.1	PDJ@0.2	PDJ@0.3	PDJ@0.4
Nose	100	100	100	100
Eye Inner	100	100	100	100
Eye	100	100	100	100
Eye Outer	100	100	100	100
Ear	0	0	0	0
Mouth	100	100	100	100
Shoulder	100	100	100	100
Elbow	**50**	**100**	100	100
Wrist	**0**	**100**	100	100
Pinky	**0**	**0**	**0**	**100**
Index	**0**	**0**	**100**	100
Thumb	0	0	0	0
Hip	**50**	**100**	100	100
Knee	100	100	100	100
Ankle	100	100	100	100
Heel	**50**	**100**	100	100
Foot Index	0	0	0	0

**Notes.**

(Significance of Bold Text): The Wrist, Pinky and Index body parts improvise PDJ rate from 0% to 100% at different PDJ level. The Elbow, Hip and Heel body parts improvise PDJ rate from 50% 100% at different PDJ level. (Significance of Red Text): The Ear, Thumb and Foot Index body parts indicate PDJ rate of 0% due to invisibility of these body part in Downdog Yoga Pose.

**Figure 12:**
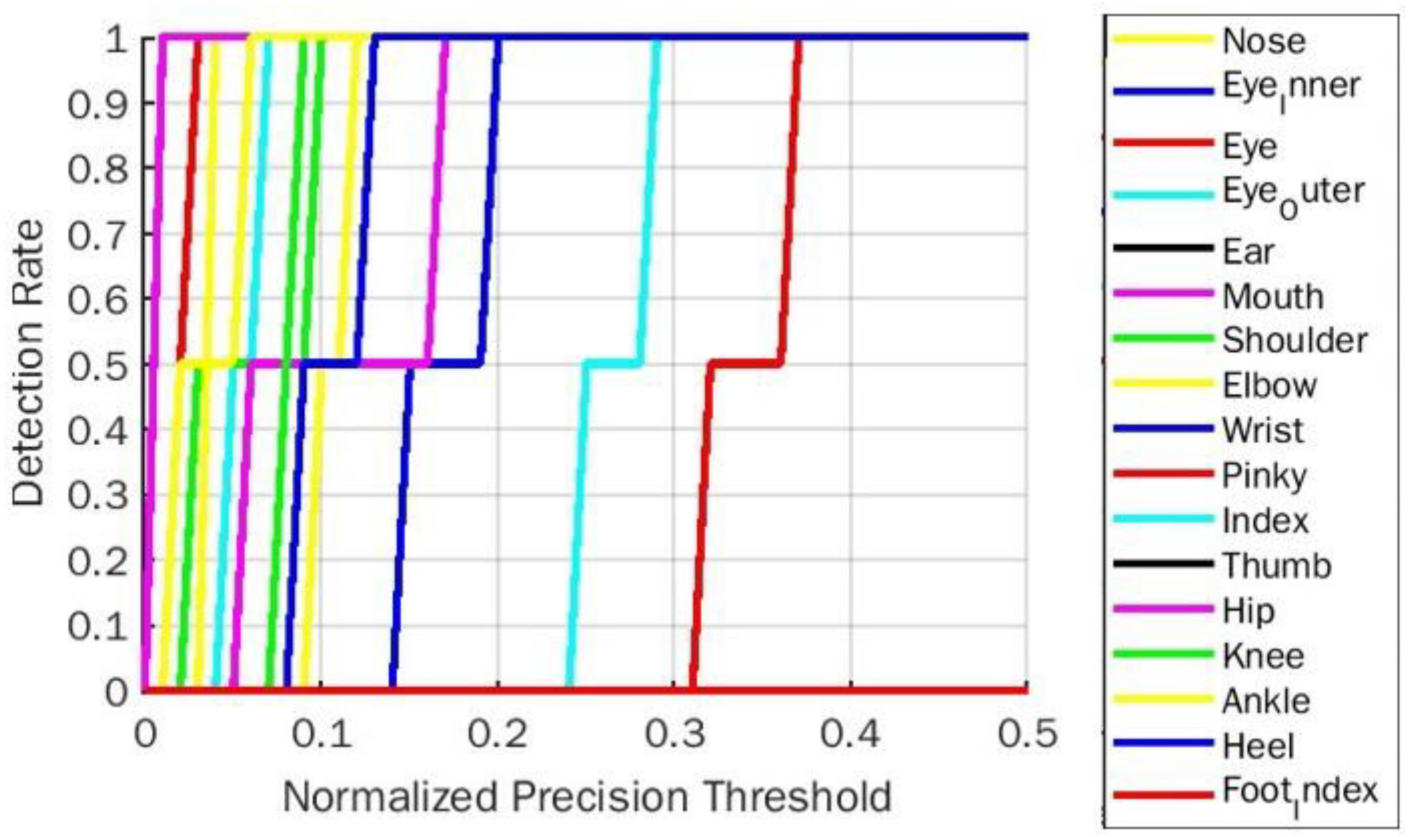
PDJ0.1_0.4 detection rate on downdog yoga pose for BlazePose.

The downdog yoga pose achieves minimum PDJ among all other yoga poses. This is because, in the downdog yoga pose, the missing ground truth body joints are replicated with the visible ground truth body joints considering symmetry. Table 8 shows PDJ rates for the downdog yoga poses for various body parts. Figure 12 shows the PDJ0.1 →0.4 detection rate on downdog yoga pose for the BlazePose model.

Table 9 represents the PDJ evaluation achieved for various yoga poses using the BlazePose model. Here the average PDJ (PDJ0.1 → 0.4) is considered for individual body parts for respective yoga poses. Quantitative information described in Table 9 shows that the overall body pose is achieved by estimating several body parts from Nose to Foot Index. As shown in the table, the goddesss pose achieved maximum PDJ, *i.e.,* 89.71%, whereas the downdog pose obtained minimum PDJ, *i.e.,* 25.74%. Among all the yoga poses, the downdog pose and the plank pose achieved minimum PDJ accuracy because the model identifies few of the body parts are absent or over imposing in both the yoga poses.

**Table 9:** Individual percentage of detected joints (PDJ) on various yoga poses using BlazePose model.

**Body parts**	**Yoga poses**				
	**Goddess**	**Tree**	**Warrior2**	**Plank**	**Downdog**
Nose	100	100	100	100	50
Eye Inner	100	100	50	0	25
Eye	100	100	50	50	25
Eye Outer	100	100	50	50	25
Ear	100	100	50	50	0
Mouth	100	100	50	50	25
Shoulder	100	100	100	50	25
Elbow	62.5	100	100	50	25
Wrist	100	100	100	50	25
Pinky	75	87.5	87.5	50	18.75
Index	75	0	75	37.5	18.75
Thumb	75	0	100	50	0
Hip	37.5	75	75	50	25
Knee	100	100	87.5	50	50
Ankle	100	100	100	50	50
Heel	100	50	50	50	50
Foot Index	100	50	50	50	0
**Mean**	**89.71**	80.15	75.00	49.26	25.74

**Notes.**

(Significance of Bold Text): The Goddess Yoga Pose achieved highest mean PDJ rate as 89.71% among all other Yoga Poses.

**Figure 13:**
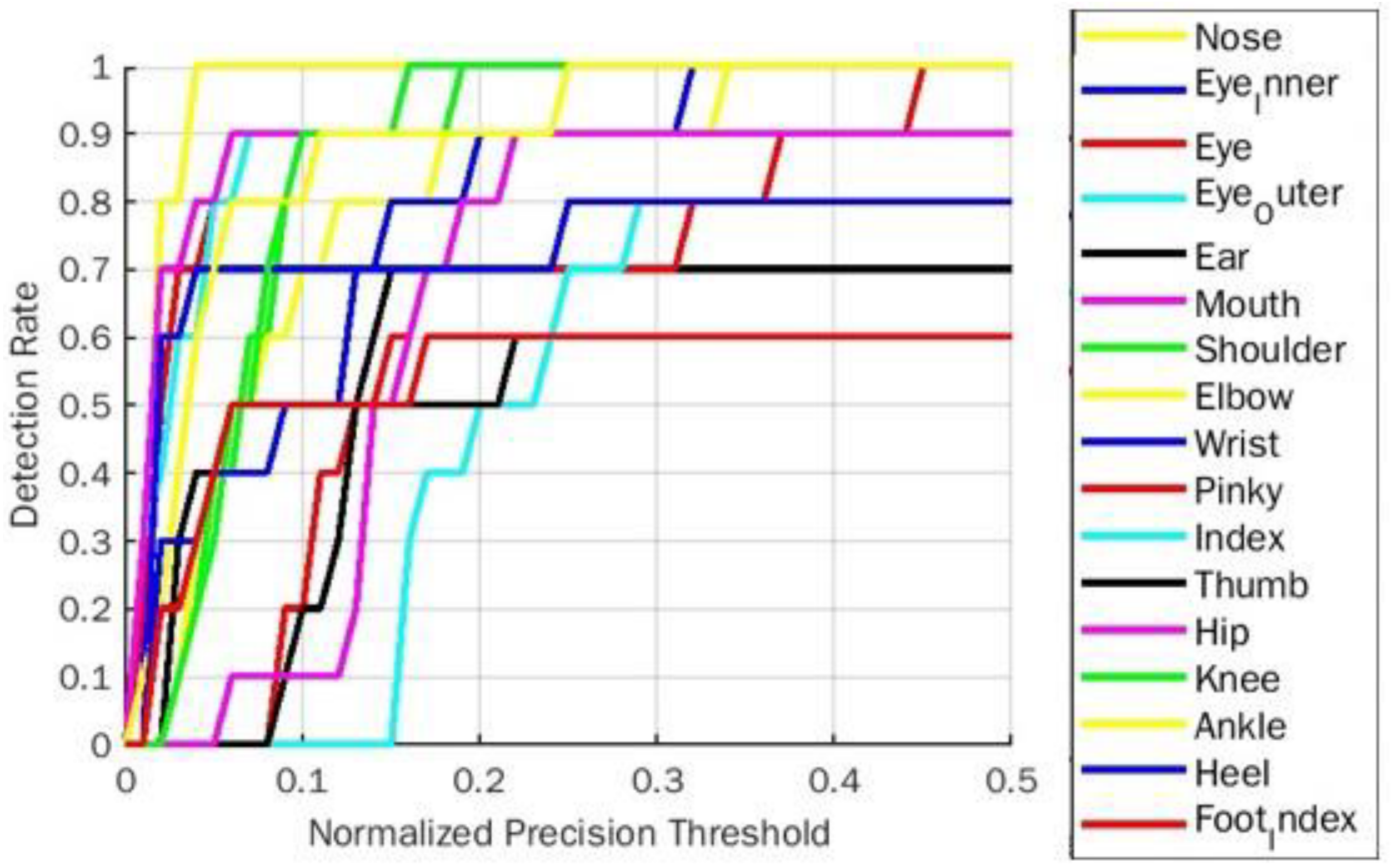
Overall PDJ0.1_0.4 detection rate on all five yoga poses for BlazePose.

**Figure 14:**
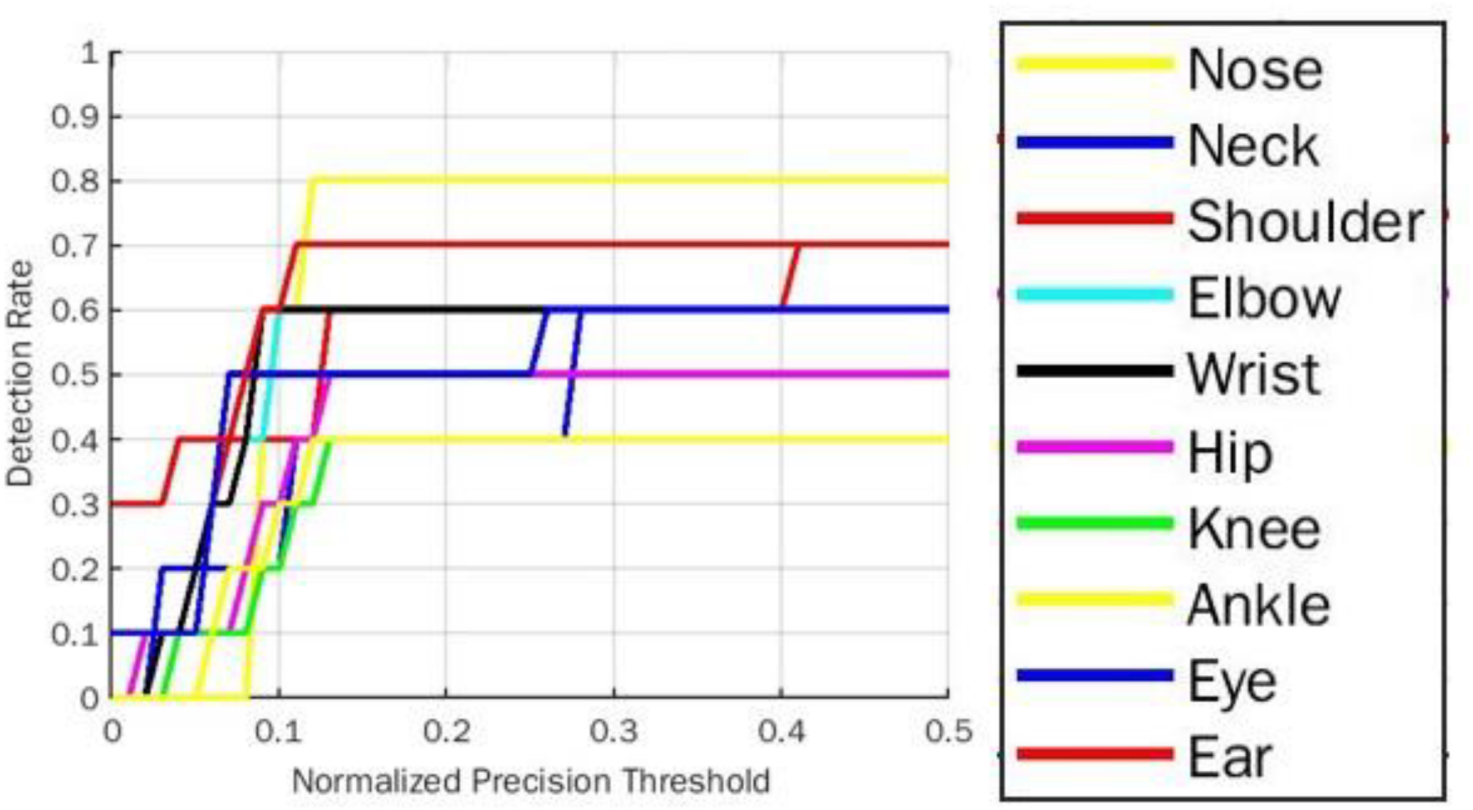
Overall PDJ0.1_0.4 detection rate on all five yoga poses for OpenPose.

The PDJ0.1 →0.4 comparative analysis for all five yoga poses for BlazePose and OpenPose model is represented in Table 10. For both models, PDJ parameters improvise their results in staggering mode from PDJ0.1 to PDJ0.4. It shows that PDJ0.4 is maximum in the range of 70% to 100% for the BlazePose model for common body parts compared to the OpenPose model. However, the PDJ0.4 for ear body parts for the BlazePose model is the same as the OpenPose model. This is because ear key points for downdog and plank yoga poses are almost missing in the ground truth localization of BlazePose joints. So the missing localization of the respective body part is replicated with symmetry body joint location of the same pose. Figures 13 and 14 show an overall PDJ0.1 →0.4 detection rate on all five yoga poses for BlazePose and OpenPose, respectively.

**Table 10:** Comparative analysis of PDJ 0.1–>0.4 with other model for yoga dataset.

**Model name**	**PDJ points**	**Common body parts**								
		**Nose**	**Eye**	**Ear**	**Shoulder**	**Elbow**	**Wrist**	**Hip**	**Knee**	**Ankle**
BlazePose	PDJ@0.1	**100**	**90**	50	**90**	**70**	**70**	10	**90**	**80**
OpenPose	PDJ@0.1	60	50	**60**	40	60	60	**30**	20	30
BlazePose	PDJ@0.2	**100**	**90**	**70**	**100**	**90**	**90**	**80**	**100**	**90**
OpenPose	PDJ@0.2	80	50	**70**	60	60	60	50	40	40
BlazePose	PDJ@0.3	**100**	**90**	**70**	**100**	**90**	**90**	**90**	**100**	**100**
OpenPose	PDJ@0.3	80	60	**70**	60	60	60	50	40	40
BlazePose	PDJ@0.4	**100**	**90**	**70**	**100**	**100**	**100**	**90**	**100**	**100**
OpenPose	PDJ@0.4	80	60	**70**	60	60	60	50	40	40

**Notes.**

(Significance of Bold Text): The BlazePose Model achieve highest PDJ rate at all PDJ level for common body parts as compared to the OpenPose Model.

Comparative analysis of PCK evaluation parameters for MPII dataset of existing model viz ConvNet (Carreira et al., 2016), Stacked Hour Glass (Newell, Yang & Deng, 2016) & HrNet (Sun et al., 2019) along with BlazePose model (with yoga pose dataset) is demonstrated in Table 11. It is observed that all existing models are targeted for the minimum number of body joints like shoulder, elbow, wrist, *etc*. In contrast, the BlazePose model can detect the maximum number of body joints. It is also observed that the BlazePose model for the yoga pose dataset gives higher accuracy for almost all the body joints than the other models with the MPII dataset. It shows that the proposed approach provides the highest accuracy for complex datasets.

**Table 11:** Comparative analysis of PCK with other models and different dataset.

**Model name**	**Dataset**	**Common body parts**								
		**Nose**	**Eye**	**Ear**	**Shoulder**	**Elbow**	**Wrist**	**Hip**	**Knee**	**Ankle**
ConvNet	MPII	–	–	–	91.9	83.9	77.8	80.9	72.3	64.8
Stacked Hour Glass	MP-II	–	–	–	96.3	91.2	87.1	90.1	87.4	83.9
HrNet	MP-II	–	–	–	96.9	92.8	89.0	91.5	89.0	85.7
**BlazePose**	**Yoga Pose**	100	90	70	**100**	**90**	**90**	**80**	**100**	**90**

**Notes.**

(Significance of Bold Text): The BlazePose Model with Yoga Pose dataset achieve highest PCK rate for all common body parts as compared to other existing models with MPII dataset.

**Table 12:** Body joint accuracy analysis and its comparison for various poses.

Name of yoga pose	Number of joints targeted	Precision	Recall	F1-score	Overall accuracy
Downdog	17	0.97	0.99	0.98	88%
Goddess			0.82	0.70		0.76	
Plank			0.98	0.94		0.96	
Tree			0.82	0.88		0.85	
Warrior2		0.80	0.87	0.83	
Downdog	33	0.98	0.99	0.98	91%
Goddess			0.80	0.77		0.78	
Plank			0.98	0.96		0.97	
Tree			0.84	0.88		0.85	
Warrior2		0.83	0.84	0.84	

**Figure 15:**
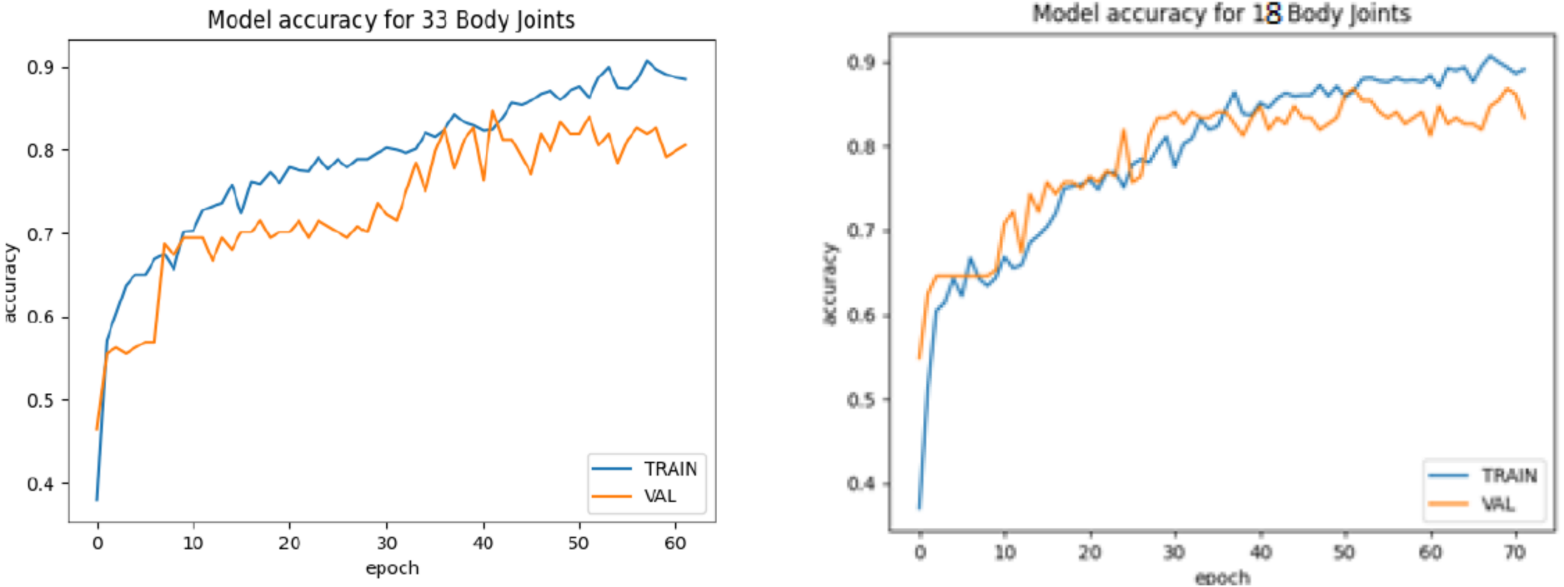
Model accuracy curve. (A) For 33 body joints. (B) For 18 body joints.

**Figure 16:**
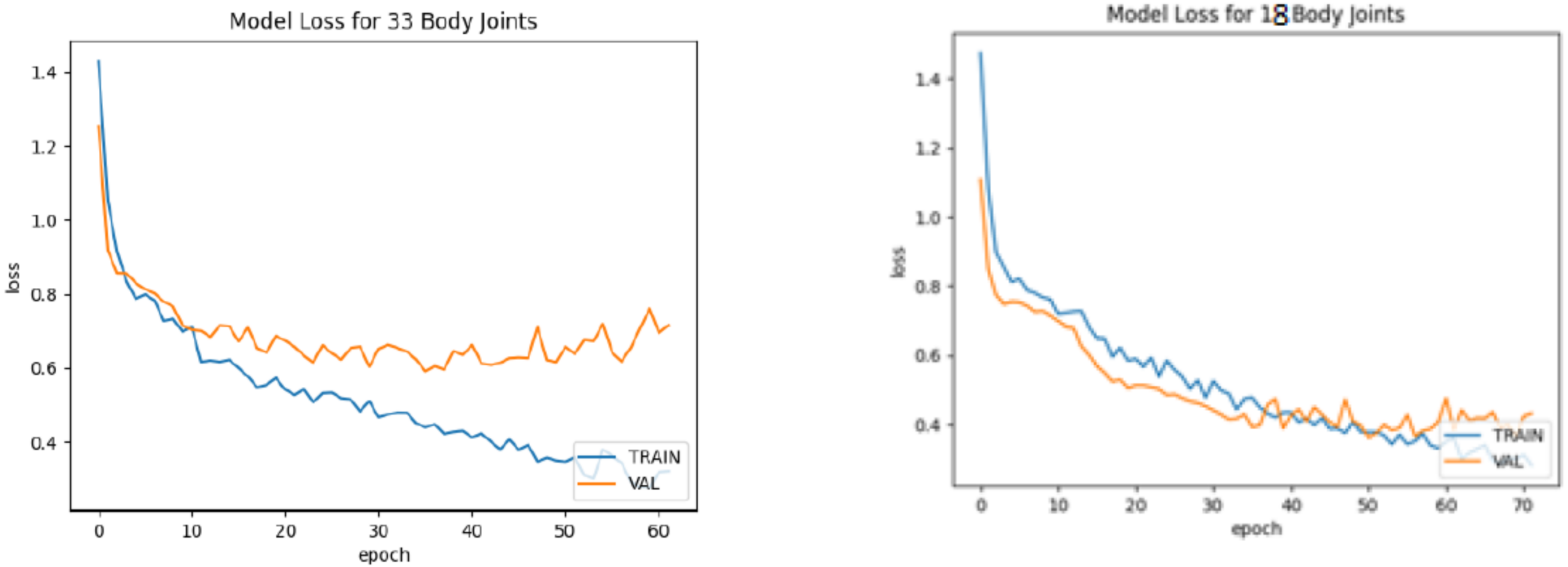
Model loss curve. (A) For 33 body joints. (B) For 18 body joints.

The bifurcation of training and testing images of the yoga pose dataset is further used to train the BlazePose model. The model is trained individually for the training and testing image sets. Furthermore, the model is evaluated with different evaluation parameters: accuracy, loss, precision, recall, and f1-score individually for 33 and 18 body joints. Figures 15(A) and Fig. 15(B) demonstrate the model accuracy curve for 33 body joints and 18 body joints. The model loss curve for 33 and 18 body joints is represented in Fig. 16(A) and Fig. 16(B). Table 12 demonstrates the additional model parameters, viz. Precision, recall, and F1-score for 33 and 18 body joints. It is observed that the model achieved 91% accuracy in localizing the maximum body joints.

## Conclusions

This article presents a different approach to human body joint detection and its analysis for human pose estimation accuracy. The proposed method is demonstrated in the five different yoga poses. Body joints are detected using the BlazePose model—a machine learning pipeline approach. The model sees the maximum number of body joints (33) to estimate the overall pose. The accuracy of detected body joints is represented with PCK and PDJ evaluation parameters. PCK and PDJ measurement algorithms evaluate the distance between the predicted and actual joint locations. According to the test results, the model successfully detected almost all body joints except downdog pose and the plank pose. The posture has half-body visibility in the downdog and plank poses; therefore, half of the body joints cannot be detected. These missing joints affected the accuracy. Usually, the missing body joints are at the same location as the visible half-body joints. Therefore, we can justify that a 50% detection rate is equivalent to 100% for these two poses. PCK results achieved a maximum of 93.9% for the goddess pose among all five poses. The PDJ results are conducted in the staggering mode as PDJ0.1 → 0.4. The proposed model succeeded with PDJ ranging from 90% to 100% for almost all the body joints. The maximum PDJ result is achieved for goddess pose as 89.71%. The proposed approach also represents a comparative analysis of PCK and PDJ parameters of the BlazePose and OpenPose models. BlazePose model recognizes 33 body joints with higher accuracy than 18 OpenPose body joints.

Furthermore, there is a significant difference in PCK and PDJ analysis of BlazePose and OpenPose models. PCK and PDJ results for BlazePose can be improved if all the body joints are detected in the downdog and plank poses. Additionally, the system can be extended by applying the proposed approach to complex yoga poses like surya namaskara.

##  Supplemental Information

10.7717/peerj-cs.1152/supp-1 Supplemental Information 1 Code Human Pose Estimation

## Additional Information and Declarations

### Data Availability

The following information was supplied regarding data availability: The raw data and code are available in the Supplemental File and at figshare: Desai, Miral (2022): Code_Human Pose Estimation.zip. figshare. Online resource. https://doi.org/10.6084/m9.figshare.21285594.v1. Third party data from Kaggle was used for the experiments:
https://www.kaggle.com/datasets/niharika41298/yoga-poses-dataset.
