# Supplementary figures and images for: A novel approach for yoga pose estimation based on in-depth analysis of human body joint detection accuracy
Source: PeerJ Comput Sci. 2023 Jan 13;9:e1152. doi: 10.7717/peerj-cs.1152 (PMC10280249; doi:10.7717/peerj-cs.1152)

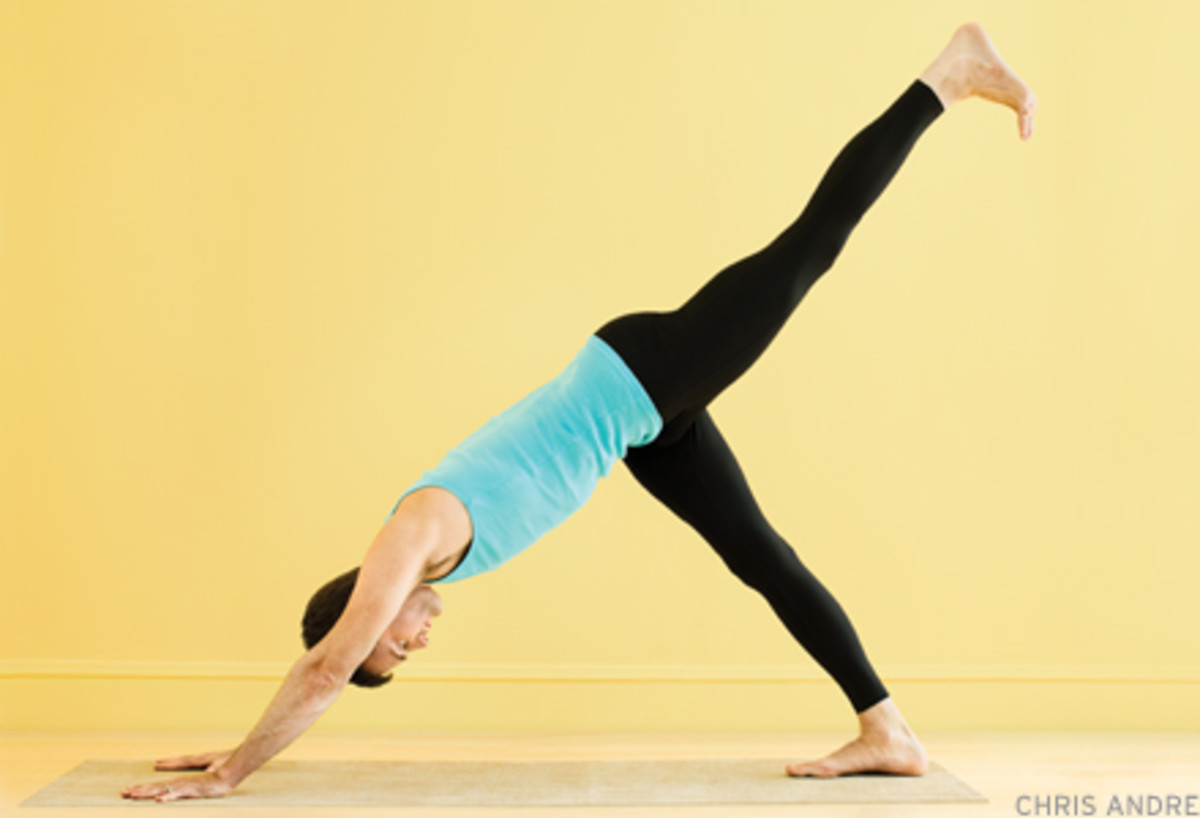

Supplement: Supplemental Information 1 [file peerj-cs-09-1152-s001.zip › Code_Human Pose Estimation/Sample Image/Downdog.jpg]

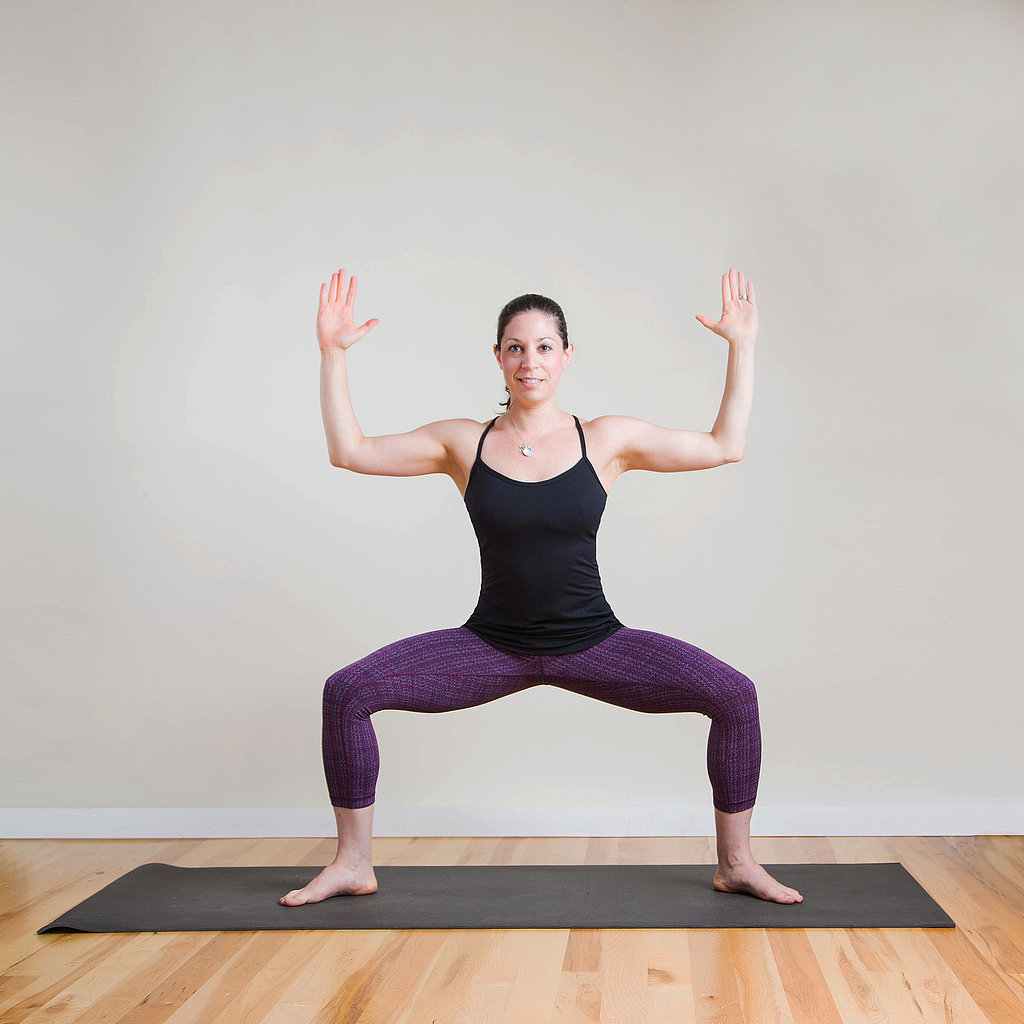

Supplement: Supplemental Information 1 [file peerj-cs-09-1152-s001.zip › Code_Human Pose Estimation/Sample Image/Goddess.jpg]

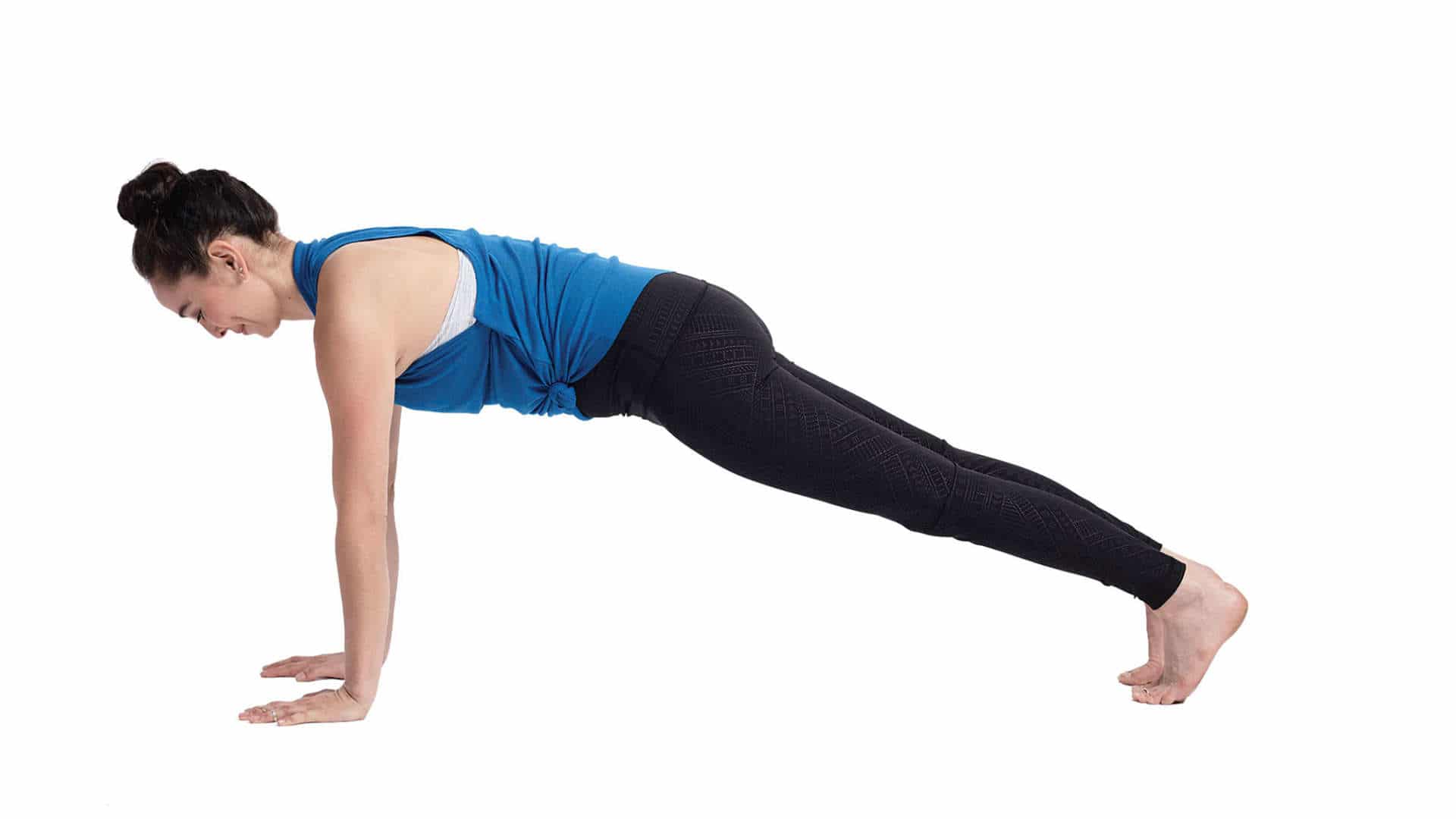

Supplement: Supplemental Information 1 [file peerj-cs-09-1152-s001.zip › Code_Human Pose Estimation/Sample Image/Plank.jpg]

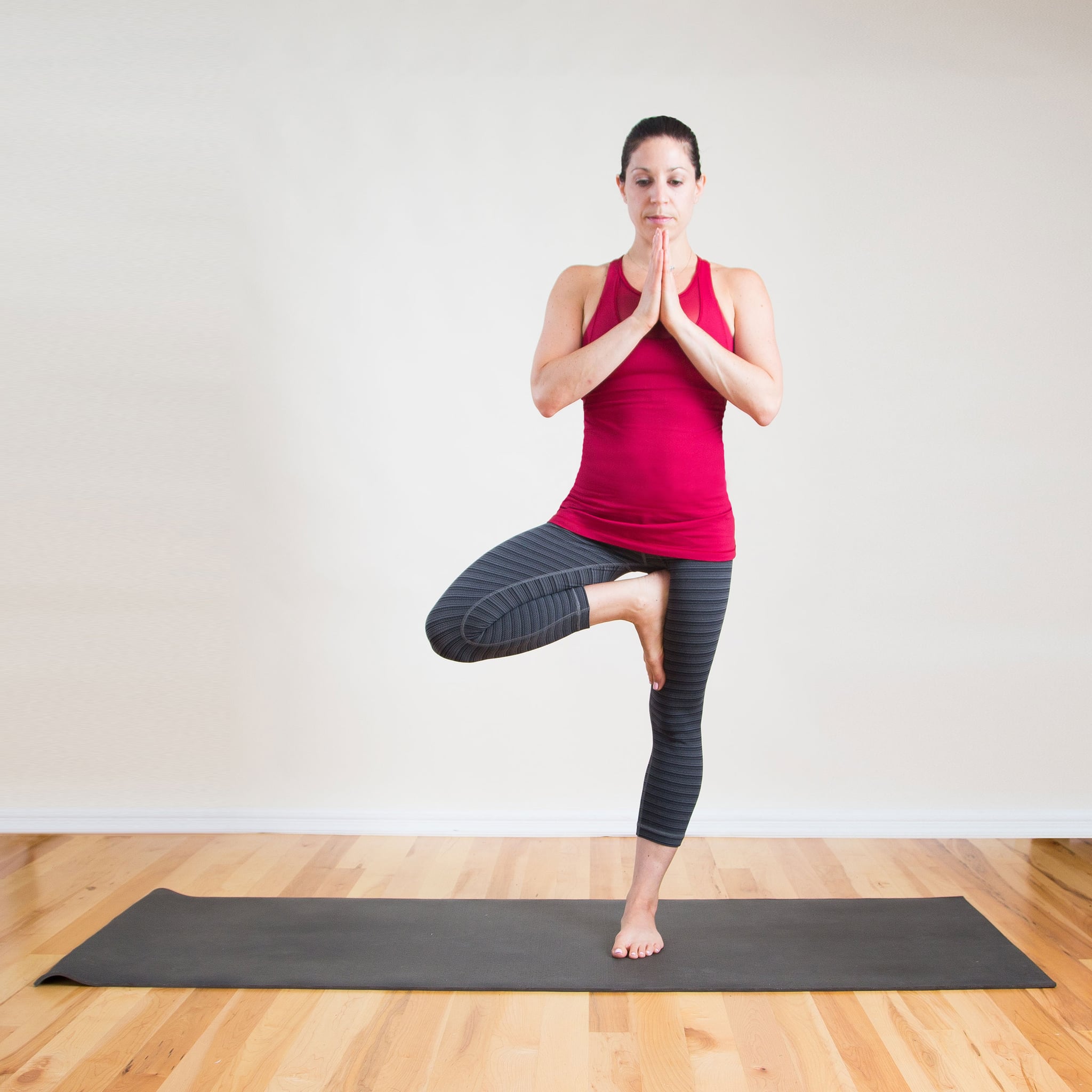

Supplement: Supplemental Information 1 [file peerj-cs-09-1152-s001.zip › Code_Human Pose Estimation/Sample Image/Tree.jpg]

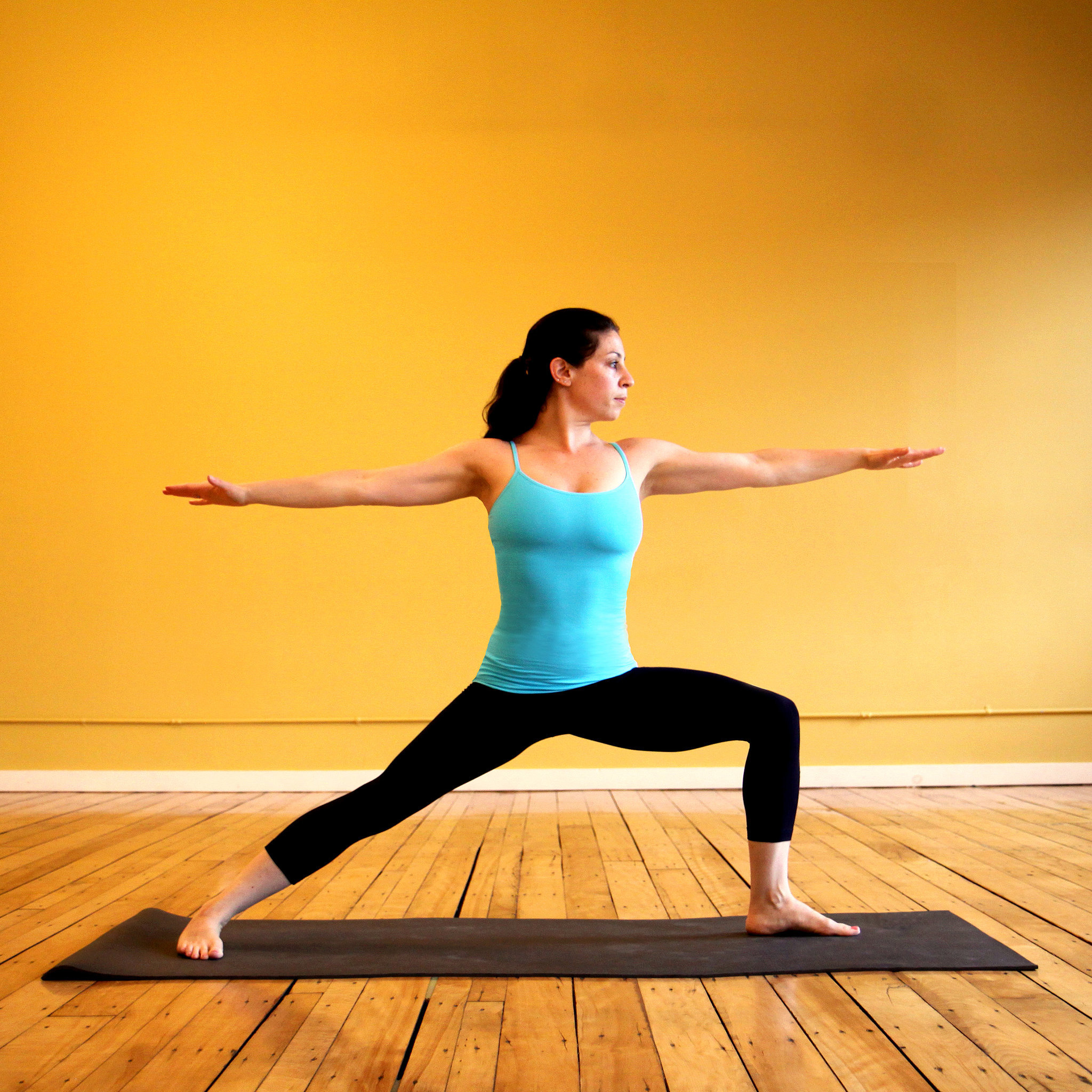

Supplement: Supplemental Information 1 [file peerj-cs-09-1152-s001.zip › Code_Human Pose Estimation/Sample Image/Warrior2.jpg]
